# Genomic dynamics of brown trout populations released to a novel environment

**DOI:** 10.1002/ece3.9050

**Published:** 2022-07-03

**Authors:** Sara Kurland, Nima Rafati, Nils Ryman, Linda Laikre

**Affiliations:** ^1^ Department of Zoology, Division of Population Genetics Stockholm University Stockholm Sweden; ^2^ Department of Medical Biochemistry and Microbiology National Bioinformatics Infrastructure Sweden, Science for Life Laboratory, Uppsala University Uppsala Sweden

**Keywords:** conservation genetics, genetic monitoring, hybridization, population translocations, whole‐genome sequencing

## Abstract

Population translocations occur for a variety of reasons, from displacement due to climate change to human‐induced transfers. Such actions have adverse effects on genetic variation and understanding their microevolutionary consequences requires monitoring. Here, we return to an experimental release of brown trout (*Salmo trutta*) in order to monitor the genomic effects of population translocations. In 1979, fish from each of two genetically (*F*_ST_ = 0.16) and ecologically separate populations were simultaneously released, at one point in time, to a lake system previously void of brown trout. Here, whole‐genome sequencing of pooled DNA (Pool‐seq) is used to characterize diversity within and divergence between the introduced populations and fish inhabiting two lakes downstream of the release sites, sampled 30 years later (c. 5 generations). Present results suggest that while extensive hybridization has occurred, the two introduced populations are unequally represented in the lakes downstream of the release sites. One population, which is ecologically resident in its original habitat, mainly contributes to the lake closest to the release site. The other population, migratory in its natal habitat, is genetically more represented in the lake further downstream. Genomic regions putatively under directional selection in the new habitat are identified, where allele frequencies in both established populations are more similar to the introduced population stemming from a resident population than the migratory one. Results suggest that the microevolutionary consequences of population translocations, for example, hybridization and adaptation, can be rapid and that Pool‐seq can be used as an initial tool to monitor genome‐wide effects.

## Graphical abstract

The consequence of human induced translocations on natural populations is monitored over c. 3 decades in wild brown trout (Salmo trutta). Two genetically distinct populations released to the same environment are unequally represented in descendant populations, with one introduced population mainly contributing to the lake closest to the release site and the other dominating waters further downstream.

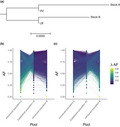

## 1 INTRODUCTION

Populations of the same species but genetically divergent backgrounds increasingly come into contact with each other and/or with new environments as habitats are altered or destroyed due to human activities, including through climate change displacing species from their native ranges (Crispo et al., 2011; Diamond, 2018; Zlonis & Gross, 2018). Populations may also enter environments where they have not occurred before and/or come into contact with genetically divergent conspecific populations through translocations, where individuals are moved from one place to another in order to establish new populations or to supplement preexisting ones (Ottewell et al., 2014; Weeks et al., 2011). Large‐scale releases of translocated, captive bred, or cultivated individuals (i.e., supplementary releases) into native populations are conducted for a variety of reasons, for example, to increase biomass for commercial and recreational harvest or to meet conservation objectives (Laikre et al., 2010; Tallmon et al., 2004).

While there are benefits to population translocations, they pose potential threats to intraspecific genetic variation (Laikre et al., 2010; Olden et al., 2004). Integration of foreign genetic material may compromise the genetic integrity of wild populations, for example, through genetic homogenization (Östergren et al., 2021; Petereit et al., 2018). Hybridization between conspecifics may increase genetic diversity which might facilitate adaptation (Meier et al., 2019; Zhang et al., 2020). However, hybridization risks maladaptation when populations of different local adaptations are mixed (Besnier et al., 2022; Jensen et al., 2017). A related avenue of inquiry regards the success of translocated individuals in the new environment. Initial levels of genetic variation within progenitor populations are predicted to determine the ability of genes to respond to new selective pressures (Vigouroux et al., 2002). Many released populations, especially captive bred ones, have experienced recent bottlenecks predicted to reduce variation (Martinez et al., 2022; Ryman & Ståhl, 1980). It is, therefore, of interest to address the ability of genetically impoverished populations to adapt over a few generations (Willoughby et al., 2018). Studying the consequences of population translocations is, thus, of relevance both for our knowledge of adaptive evolution and for sustainable management and conservation (Liddell et al., 2021; Pierce et al., 2017).

An increasing number of studies utilize high‐throughput methodologies to study genomic variation, yet the majority of them are directed at model organisms or domesticated conspecifics (Carneiro et al., 2014; Rubin et al., 2010). Many also cover large areas with strong environmental gradients, extensive time frames, and/or species with large populations, for which selection is expected to be strong (Barrio et al., 2016; Kjærner‐Semb et al., 2021). Additional characterization of intraspecific variability over small and/or environmentally homogenous areas, contemporary time frames, or within small populations subject to strong genetic drift, is warranted.

Like many salmonids, the brown trout (*Salmo trutta*) has been subjected to all of the situations raised above (Hansen et al., 2009; Valiquette et al., 2014). The brown trout is characterized by high levels of genetic substructuring and is able to maintain genetic separation over small geographic scales (Andersson et al., 2017; Ryman et al., 1979). Typically, local effective population sizes are small (Palm et al., 2003; Palmé et al., 2013). Its conservation status is under concern due to a range of anthropogenic stressors including intentional and unintended introductions (Ayllón et al., 2016). However, the genomic tools to study the effects of population translocations on brown trout intraspecific variation have been lacking (Bekkevold et al., 2020). It is only recently (2019) that an annotated brown trout reference assembly became publicly available (fSalTru1.1; https://www.ncbi.nlm.nih.gov/assembly/GCF_901001165.1/). Furthermore, this species has a large genome (c. 2.4 Gb) characterized by an ancient whole‐genome duplication c. 90 million years ago (Berthelot et al., 2014; Lien et al., 2016; Nugent et al., 2017). Studying genomic change is challenging for polyploid species where genetic drift is strong. The current study is one of the first attempts to monitor the genomic effects of population translocations for such a case over contemporary time scales.

### 1.1 Objectives

The aim of the present study is to explore the potential of using whole‐genome sequencing of pooled samples (Pool‐seq) to monitor genome‐wide diversity and divergence of brown trout following introduction into a novel environment. Two genetically and ecologically distinct populations were simultaneously released, at one point in time (in 1979), to a natural lake system void of brown trout prior to the release. Fish from two introduced populations were genetically distinguishable at a few allozyme loci and differed in traits, for example, age at maturity, reproductive and migratory behavior, and body size (Palm & Ryman, 1999). Here, samples from introduced fish are studied as well as from fish established in two lakes downstream of the release sites c. 30 years later (corresponding to c. 5 generations; Palmé et al., 2013). Four groups of fish are, thus, examined; introduced fish from each of two distinct populations and fish established in two of the lakes in the new lake system.

The following questions are addressed:

1. What are the genome‐wide levels of diversity in, and divergence between, fish originating from two distinct populations simultaneously released into a novel lake system? Can adaptive divergence be identified?
2. What are the genome‐wide levels of diversity in and divergence between fish that have established within two lakes in this novel lake system located at different distances from the release sites (<1 km and c. 6 km, respectively) and sampled 30 years (c. 5 trout generations) later?
3. Are there any signs of selection in the new environment, that is, any adaptive differences between the released fish compared to fish inhabiting two lakes downstream of the release site c. 30 years later?

## 2 MATERIALS AND METHODS

### 2.1 Study system

**FIGURE 1:**
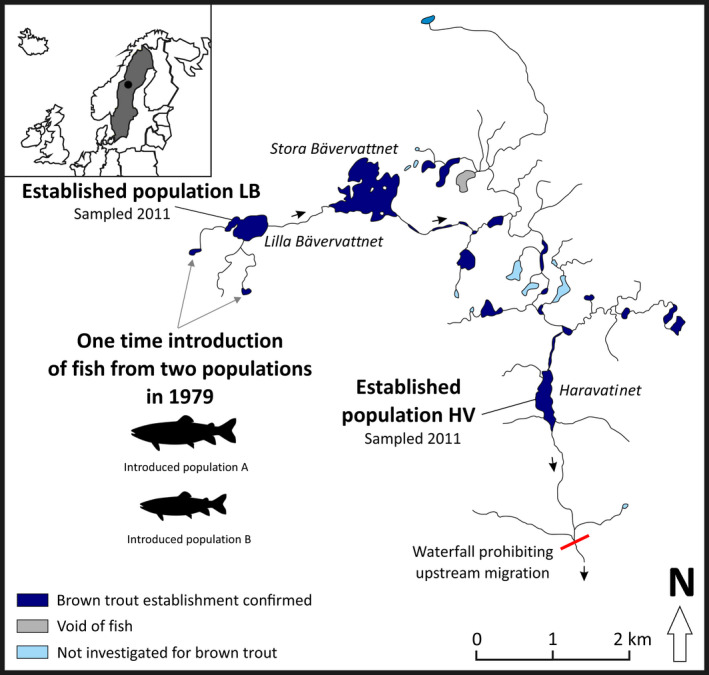
Map of the Bävervattnen lake system and sampled populations (in bold). Brown trout (*Salmo trutta*) from two distinct populations, introduced populations A and B, were simultaneously released at one point in time in 1979. Five hundred juveniles from each population were introduced in each of the two marked tarns. Fish established since the introduction were caught in Lakes Lilla Bävervattnet (established population LB) and Haravattnet (established population HV) in 2011. Lakes in which brown trout have been found since the release are dark blue, light blue lakes have not yet been investigated, and gray waters are known to be void of brown trout. Arrows indicate the direction of water flow and the red line represents a waterfall separating waters void of brown trout prior to the introduction (above the fall) from those naturally inhabited by brown trout (below the fall)

We studied brown trout originating from two genetically and ecologically distinct populations released to the same novel lake system, as well as fish established in the wild. Established fish were collected over 30 years later (corresponding to c. 5 generations; Palmé et al., 2013) in two lakes downstream of the release sites (Lakes Lilla Bävervattnet and Haravattnet; Figure 1).

The released fish originated from two populations with diverse ecological features separated by more than 500 km waterway and likely isolated from each other since the last glaciation (c. 5000–9000 years ago; Palm & Ryman, 1999). Released fish from the two populations exhibited contrasting homozygosity at one allozyme locus and were genetically divergent at other allozyme loci (Palm & Ryman, 1999). The release was carried out once, in a mountain lake system located in Hotagen in Jämtland County, central Sweden (Figure 1). In July 1979, 1000 juvenile fish (fry) from each of the two populations were released at two locations in the upstream part of the Lake Bävervattnen system (Figure 1). Five hundred fish from each introduced population were released simultaneously at each site. This water system was void of brown trout prior to the introduction and only inhabited by Arctic charr (*Salvelinus alpinus*) which have remained since.

Half of the released fish were from a hatchery population which, in turn, originates from a wild population in Lake Kallsjön, a large (c. 160 km^2^) lake located 100 km west of the study area. The wild fish that once inhabited this lake were large, slow‐growing, piscivorous, and with the capacity for long‐distance migration (Palm & Ryman, 1999). They were extirpated in the wild in the 1980s due to dams obstructing their route to spawning grounds. Fish from this source will be referred to as *introduced population A*. The other released fish originate from a population inhabiting two small (<1 km^2^) and closely connected lakes, Lake Fälpfjälltjärnarna, located c. 10 km north of the study area with similar ecological characteristics as the presently studied lake system. The fish here are non‐migratory and ecologically typical of populations inhabiting small mountain lakes in northern Scandinavia: with small body size, early maturation, and a primarily insect‐based diet (Palm & Ryman, 1999). The released fish originating from Lake Fälpfjälltjärnarna will be referred to as *introduced population B*.

Fish established in the wild in the new lake system were sampled from Lake Lilla Bävervattnet, located <1 km downstream of the release sites, and Lake Haravattnet, c. 6 km downstream. Fish from either lake will be referred to as *established population LB* and *established population HV*, respectively. We denote the groups of fish as populations for ease of writing but acknowledge that we do not know whether they constitute genetically distinct populations. See Appendix S1 for further details on the study system.

### 2.2 Sample collection and DNA extraction

We studied four groups of fish, that is, released fish representing each of the two distinct populations (introduced population A and introduced population B), and fish established in the wild in lakes Lilla Bävervattnet (established population LB) and Haravattnet (established population HV; Figure 1). The sample size was 50 for each group. Fish from introduced populations A and B were caught in the wild in 1988–1995 and classified to either introduced population based on their age (otolith readings) or by genotype at the allozyme marker locus (Appendix S1; Palm & Ryman, 1999). Individuals representing the parental generation (*P*) or the *F1* generation for which population assignment was possible using the allozyme marker (no hybrids) are included. Fish established in Lakes LB and HV were caught in 2011.

Genomic DNA was extracted from muscle tissue from 50 individuals from each investigated group (introduced population A, introduced population B, established population LB, and established population HV) using a KingFisher cell and tissue DNA kit (Thermo Scientific) including RNase A treatment. High‐molecular‐weight DNA from each individual was combined at equal concentrations for each population in order to create pools of individuals corresponding to each population to be sequenced. Additional details about DNA extraction are provided in Appendix S1.

### 2.3 Library construction and sequencing

Samples were sent to the National Genomics Infrastructure (NGI) at the Science of Life Laboratory (SciLifeLab), Stockholm, Sweden, for the preparation of PCR‐free paired‐end libraries and sequencing (Illumina HiSeq 2000). Additional details about library construction and sequencing are provided in Appendix S1.

### 2.4 Mapping and variant calling

Illumina FASTQ files from each lane were filtered for adapters and low‐quality bases (Phred score < 20) using BBDuk implemented in BBTools v.37.31 (http://sourceforge.net/projects/bbmap/). Trimmed reads were mapped per lane to the brown trout assembly (comprising 2,371,863,509 bp; https://www.ncbi.nlm.nih.gov/genome/31807?genome_assembly_id=571197) using BWA mem v.0.7.17 (Li & Durbin, 2009). Mapped reads were sorted and merged for each population, and only properly mapped pairs were retained using SAMtools v.1.8 (Li et al., 2009). Variant calling was conducted in SAMtools, using minimum base and mapping quality scores of 20 to reduce false variant sites caused by misalignments, resulting in one mpileup file for all four pools. The “identify‐genomic‐indel‐regions.pl” script of PoPoolation2 v.1.201 (Kofler, Pandey, et al., 2011) was used to omit indels along with error‐prone 5 bp windows upstream and downstream of each indel. Pool‐seq data are sensitive to sequencing errors and variation in coverage, for example, low coverage regions overrepresented by few individuals (Kofler, Orozco‐terWengel, et al., 2011). For polyploid species, for example, the brown trout, paralogous regions are expected to have high coverage. In order to eliminate artefactual results caused by coverage fluctuations, the mpileup was subsampled based on the mode of the read depth histogram for each pool, to 20–150× using the “subsample‐pileup.pl” script implemented in PoPoolation v.2.2 (Kofler, Orozco‐terWengel, et al., 2011) in accordance with best practices for polyploid species (Micheletti et al., 2018; Narum et al., 2013) and as previously conducted for brown trout (Kurland et al., 2019; Saha et al., 2022). Additional details on quality assessment, window sizes, quality filtering, and parameter settings are described in Appendix S1.

### 2.5 Genomic variation

We examined genome‐wide diversity in introduced and established populations. Nucleotide diversity (*π*; Charlesworth & Charlesworth, 2010) and Tajima's *D* (*T*_*D*_; Tajima, 1989) were estimated using the “variance‐sliding.pl” script of PoPoolation v.2.2 (Kofler, Orozco‐terWengel, et al., 2011) in 5 kb non‐overlapping windows. See Appendix S1 for further details on parameter settings and Table S13 for the full glossary.

Allele frequencies were calculated per variant site (SNP) using the “snp‐frequency‐diff.pl” script in PoPoolation2 v1.201 (Kofler, Pandey, et al., 2011) and reformatted to reflect the number of reads corresponding to the most (major) and least (minor) abundant alleles (*n*_MAJ_ and *n*_MIN_) across all populations using a custom script (available upon request). Pooled heterozygosity score (*H*_P_) was calculated according to Rubin et al. (2010). Since PoPoolation 2 has no option to estimate allele frequencies within windows, allele frequencies and *H*_P_ were calculated per variant site. 5 kb windows were constructed using an R script (https://github.com/nimarafati/R_scripts/blob/master/Window_average.Rscript), only retaining windows with at least 33 variant sites (the average number of variant sites found per window in PoPoolation2).

### 2.6 Population differentiation

Population differentiation was estimated using *F*_ST_ from the “fst‐sliding.pl” script in PoPoolation2 v.2.2 (Kofler, Pandey, et al., 2011) within non‐overlapping windows of 5 kb to minimize stochastic errors linked to small window sizes (Kofler, Pandey, et al., 2011; Saha et al., 2022). *F*_ST_ was estimated using the default, Nei's (1973), approach, as well as one provided by Karlsson et al. (2007), which is expected to return estimates more in line with those from individually genotyped SNPs (i.e., Weir & Cockerham, 1984; cf. Saha et al., 2022, their Appendix S4).

We expect genetic change acting across the short time frame studied here to result in subtle allele frequency shifts, which may be overlooked by *F*_ST_ estimates. Pairwise differences in frequency of the most common allele across all four pools (the major allele) were, therefore, included (*ΔAF*; Carneiro et al., 2014). *ΔAF* was estimated within 5 kb windows, including all possible pairwise comparisons between pools.

Genetic distances between populations were also examined by creating a dendrogram in TreeMix (Pickrell & Pritchard, 2012). This statistical framework uses maximum likelihoods to describe distance‐based relationships between populations in bifurcating trees.

This program uses as input allele frequencies estimated in PoPoolation 2 v.2.2 (Kofler, Pandey, et al., 2011) reformatted to reflect *n*_MAJ_ and *n*_MIN_ using a custom script (available upon request) as described above. We did not include any migration edges for these four groups of fish.

### 2.7 Functional impact of divergence

Since introduced populations A and B originate from ecologically divergent populations (source populations inhabiting lakes Kallsjön and Fälpfjälltjärnarna, respectively), we characterize the functional differences between the two, in an attempt to give a more detailed description of divergence than provided by *F*_ST_ alone. Functional elements of SNPs were examined for enrichment at high allele frequency differences (*ΔAF*) as would be expected under directional selection on many independent mutations (Carneiro et al., 2014). SnpEff v.5.0 (Cingolani et al., 2012) was used to annotate the genomic distribution of variant sites and to classify them into functional elements (non‐synonymous and synonymous coding sequences, untranslated region (UTR), 5 kb upstream, 5 kb downstream, intragenic, and intergenic, following Barrio et al. (2016) and further described in Appendix S1 under genomic distribution of SNPs). For each of these functional categories, the allele frequency differences between introduced populations A and B were sorted into bins (10 equally large bins of *ΔAF* = 0–0.1, *ΔAF* = 0.1–0.2, etc.). *M*‐values for log2 fold change were retrieved by comparing the observed and expected number of SNPs per category and bin (Appendix S1; genomic distribution of SNPs). *M*‐values show a relative abundance of SNPs in a given ΔAF bin with different functional annotation. Positive values indicate that the observed frequency is larger than expected under neutrality, whereas negative values indicate observed frequency to be less than expected. Statistical significances of deviations observed from expected SNP counts were tested with standard *χ* ^2^‐tests of independence between the observed and expected number of SNPs per functional category and bin (df = 1). Thresholds for significant enrichment included *M* > 0.05 and *p* < 2.5 × 10^−11^ (corresponding to *α* = .05 corrected for multiple testing across a genome size of 2 Gb (Barrio et al., 2016; Pruisscher et al., 2018).

The same procedure of identifying the functional impact of markedly divergent SNPs was performed for the two populations established populations LB and HV, in order to characterize genomic divergence in the new lake system.

### 2.8 Adaptive divergence between introduced populations A and B

Two approaches were used to explore potential indications of adaptive differences between introduced populations A and B. First, we considered *ΔAF* between them, estimated per variant site (1 bp) and per 5 kb windows (the latter used for visualization). For the SNP‐based approach, analysis was restricted to SNPs of marked *ΔAF* (95th percentile of *ΔAF*; *ΔAF *≥ 0.73) exhibiting significant allele frequency difference between introduced populations A and B as tested by Fisher's exact test implemented in PoPoolation2 v.2.2 (Kofler, Pandey, et al., 2011) using significant threshold *p* < 2.5 × 10^−11^ (corresponding to *α* = .05 corrected for multiple testing across a genome size of 2 Gb; Pruisscher et al., 2018). These SNPs were categorized by functional impact and those resulting in non‐synonymous changes were marked as candidate SNPs for adaptive divergence between stocks.

Second, estimates of divergence and diversity were combined in order to avoid confounding selection with drift in regions of elevated divergence (Kjærner‐Semb et al., 2016). To further limit false outliers, we employed a window‐based approach in contrast to SNP‐based one (Keehnen et al., 2018; Kofler, Pandey, et al., 2011). Candidates for adaptive divergence between introduced populations were identified from independent 5 kb windows of marked differentiation between introduced populations A and B that simultaneously showed low levels of nucleotide diversity (*π*) within both introduced populations (cf. Carneiro et al., 2014; Kjærner‐Semb et al., 2016; Van Doren et al., 2017). The approach aimed to identify regions where selection has acted within both introduced populations A and B, as indicated by a reduction in *π*, but along different trajectories for each introduced population, as indicated by high *F*_ST_. The requirement to identify putatively adaptive windows included that the average *F*_ST_ within the window exceeded 0.44 (above 95th percentile of *F*_ST_) and average *π* for the same window to be below 0.44 × 10^−6^ in introduced population A and below 0.90 × 10^−6^ in introduced population B (below 5th percentile of *π* within each population pool, respectively). These thresholds were chosen in order to capture outliers in both *F*_ST_ and *π* distributions.

Genes in putatively adaptive regions (identified by *F*_ST_ and *π*) were obtained by considering non‐synonymous SNPs found within the candidate windows, only including SNPs exhibiting significant allele frequency difference between introduced populations A and B as tested by Fisher's exact test in Popoolation2 v.2.2 (Kofler, Pandey, et al., 2011). The threshold for significance used was *p* < 2.5 × 10^−11^, (corresponding to *α* = .05 corrected for multiple testing across a genome size of 2 Gb; Pruisscher et al., 2018). Genes surrounding such SNPs served as candidates for adaptive divergence in the introduced populations’ native environments. Allele frequencies within these genes were sought in the established populations LB and HV, in order to track the fate of putatively adaptive SNPs in the new environments.

### 2.9 Novel selection in the new lake system

To explore the novel selective pressures, the introduced fish may have experienced since their release into the new lake system, we scanned the genome for a high degree of fixation in either introduced or established populations. We hypothesize two selective scenarios: recent adaption from standing variation through directional selection (Barrett & Schluter, 2008) and relaxed selection (Lahti et al., 2009). Firstly, fish introduced into the new lake system may experience novel selection, acting either on preexisting variation or new mutations (Barrett & Schluter, 2008). Novel mutations are disregarded in the present study, as the probability for mutation is small over so few generations (Ryman & Leimar, 2008). Instead, we sought SNPs shaped by directional selection acting on standing variation, characterized by reduced variation in established populations LB and HV in comparison to introduced populations A and B.

In the second scenario, selection in the new lake system may be relaxed in comparison to the introduced populations' native environments. If so, genomic regions which were under selective constraint in the source environment may accumulate genetic variation in the new lake system. This may be reflected in regions of increased variation in the established populations compared to the introduced populations. However, the possibility for hybridization to have also shaped these regions cannot be excluded.

Candidates of directional and relaxed selection were sought by using normalized heterozygosity scores within each pool (*ZH*_P_) and comparing contrasting population pairs: introduced populations A and B compared to established populations LB and HV. Candidates for directional selection were characterized as 5 kb windows of *ZH*_P_ below the genome‐wide average within each of established populations LB and HV and *ZH*_P_ above the genome‐wide average within each of introduced populations A and B. Candidates for relaxed selection are defined to have *ZH*_P_ below the genome‐wide average within each of the introduced populations A and B and *ZH*_P_ above the genome‐wide average within each of the established populations LB and HV. *ZH*_P_ was used since its distribution is characterized by *μ* = 0 and *σ* = 1. Any deviation in *H*_P_ from the pool mean is, thus, equivalent to *ZH*_P_ above or below 0 (further details in Appendix S1). Candidates for directional selection were, thus, defined as *ZH*_P_ < 0 within each of the established populations and *ZH*_P_ > 0 within each of the introduced populations. Candidates for relaxed selection were defined to have *ZH*_P_ < 0 within each of the introduced populations A and B and *ZH*_P_ > 0 within each of the established populations. Additional thresholds were applied in order to restrict analyses to windows representing the extreme lower ends of the *ZH*_P_ distributions within either population pair. *ZH*_P_ < −2 within both established populations was used as a cutoff for candidates of directional selection and *ZH*_P_ < −4 within both introduced populations for windows shaped by relaxed selection (Figure S1).

This approach of contrasting population pairs has the added benefit of lessening the problem with fixation due to drift, wherein drift and inbreeding in a population of restricted size can cause a reduction in heterozygosity over large chromosomal segments (cf. Kardos et al., 2015; Kjærner‐Semb et al., 2021; Rubin et al., 2010; Willoughby et al., 2018). The probability of genetic drift resulting in reduced heterozygosity in the same region in more than one population simultaneously, as in our search for selection in the new lake system, is presumably small (Kjærner‐Semb et al., 2021).

R‐studio v.4.0.3 (R Core Team, 2020) was used for all statistical testing and visualization (using ggplot2; Wickham, 2016). The significance threshold was *p* < 2.5 × 10^−11^, corresponding to *α* = .05 corrected for multiple testing across a genome size of 2 Gb (Pruisscher et al., 2018).

## 3 RESULTS

### 3.1 Mapping and variant calling

Sequencing led to an average of 190 Gb per population pool and average depth of coverage of 80×. Further information on data quality and mapping success is presented in Table S1. The mpileup containing reads from all four population pools has c. 1.8 × 10^9^ variant sites. Of these, between 6 and 11 M biallelic SNPs were within coverage 20–150× and mapped to chromosomes (no orphans), and these are used for estimating various population genomic parameters (Table S2).

### 3.2 Patterns of genomic variation

**FIGURE 2:**
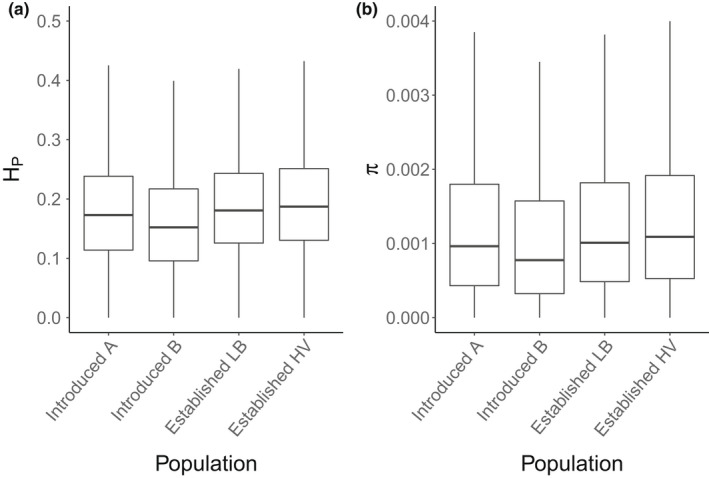
Genome‐wide diversity per population pool. Boxplots of (a) heterozygosity score per pool (*H*_P_) and (b) nucleotide diversity (*π*) estimated across 5 kb windows. Each box provides a median value, the lower (0.25) and upper quartile (0.75), and whiskers indicate a range of observed values. Results from two sample *t*‐tests and Wilcoxon tests of equality of means of *H*_P_ and *π* are significant for all pairwise comparisons (Tables S4 and S5)

All pairwise tests of nucleotide diversity (*π*), pooled heterozygosity score (*H*_P_), and Tajima's *D* (*T*_*D*_), show significant differences between populations (Tables S4–S6). Genome‐wide variation—*π* and *H*_P_—is lower in introduced populations A and B than in the established populations. Introduced population B in particular exhibits comparatively low values of *π* and *H*_P_ (Figure 2, Table S3, Figures S1 and S2). When comparing established populations in Lakes Lilla Bävervattnet (LB) and Haravattnet (HV), HV shows higher genetic diversity than the established population LB (Figure 2, Table S3, Figure S1). Genome‐wide *T*_*D*_ is slightly positive in introduced population A and established population HV and slightly negative in both introduced population B and established population LB, indicting excess of rare variants within the latter two pools (Figure S1).

### 3.3 Population differentiation

**FIGURE 3:**
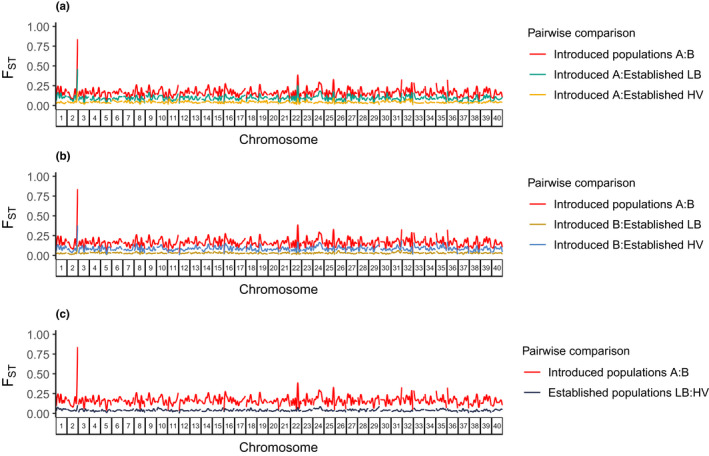
Genome‐wide differentiation (*F*_ST_) between all pairs of populations across all 40 chromosomes. Pairwise *F*_ST_ between (a) introduced population A and all other pools, (b) introduced populations B and all other pools, and (c) established populations in Lakes Lilla Bävervattnet (LB) and Haravattnet (HV). *F*_ST_ was estimated within 5 kb windows

**FIGURE 4:**
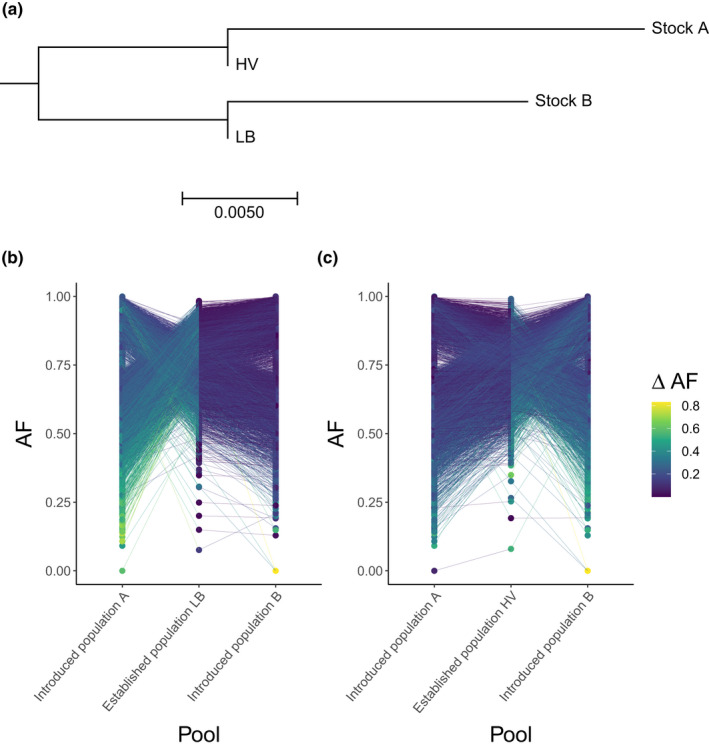
Genetic relationships between the four populations (a) across the full genome and (b, c) regions of strong allele frequency difference between introduced populations. (a) Distance‐based dendrogram estimated from allele frequencies in TreeMix (Pickrell & Pritchard, 2012), where the scale indicates the proportion of genetic divergence per unit length of the branch. Relationship between established populations (b) LB and (c) HV to each of the introduced populations A and B for 5 kb windows of a marked difference in allele frequency (*ΔAF*) between introduced populations (above 95th percentile of *ΔAF*; *ΔAF* ≥ 0.26; 8739 windows). Each dot corresponds to a window in the genome where lines connect windows and are colored by ΔAF between each established population and introduced populations, respectively

Of all possible pairwise comparisons, we find the highest divergence between the two introduced populations A and B (*F*_ST_ = 0.16) and indications of difference in the degree of genetic contribution from the introduced populations A and B to each of the established populations (Table 1; Figure 3a–c). Introduced population B shows more genetic similarity to established population LB (*F*_ST_ = 0.04) than to HV (*F*_ST_ = 0.09), while introduced population A appears to have contributed more to HV (*F*_ST_ = 0.04) than to established population LB (*F*_ST_ = 0.10). Differentiation between established populations HV and LB averages *F*_ST_ = 0.04. The dendrogram of genetic relationships among all four populations has two major branches with introduced population A and established population HV on one and introduced population B and established population LB on the other (Figure 4a). The distance between introduced population A and established population HV is somewhat greater than the distance between introduced population B and established population LB.

**TABLE 1:** Population divergence given as average and median, genome‐wide *F*_ST_ between all pairwise comparisons of population pools estimated across 329,853 windows 5 kb in size, corresponding to 11,007,131 variant sites. The default Nei's *F*_ST_ (1973) of PopPoolation2 (Kofler, Orozco‐terWengel, et al., 2011) was used in subsequent analyses, but the approach of Karlsson et al. (2007) is also provided in PoPoolation2 and was included for comparison. Note that 95% confidence intervals are provided with four decimal integers to highlight the strong statistical support for the mean *F*_ST_ values

Pairwise comparison	Nei's *F*_ST_		Karlsson's *F*_ST_	
	Mean *F*_ST_ (95% CI)	Median *F*_ST_	Mean *F*_ST_ (95% CI)	Median *F*_ST_
Introduced population A: Introduced population B	0.16 (0.1640–0.1652)	0.13	0.25 (0.2511–0.2523)	0.22
Introduced population A: Established population LB	0.10 (0.0948–0.0953)	0.07	0.15 (0.1514–0.1523)	0.12
Introduced population A: Established populations HV	0.04 (0.0427–0.0429)	0.03	0.06 (0.0634–0.0638)	0.05
Introduced population B: Established population LB	0.03 (0.0302–0.0304)	0.02	0.04 (0.0427–0.043)	0.03
Introduced population B: Established population HV	0.09 (0.0857–0.0862)	0.07	0.14 (0.136–0.1367)	0.11
Established population LB: Established population HV	0.04 (0.0395–0.0397)	0.03	0.06 (0.0578–0.0582)	0.04

Abbreviations: HV, Lake Haravattnet; LB, Lake Lilla Bävervattnet (cf. Figure 1).

**FIGURE 5:**
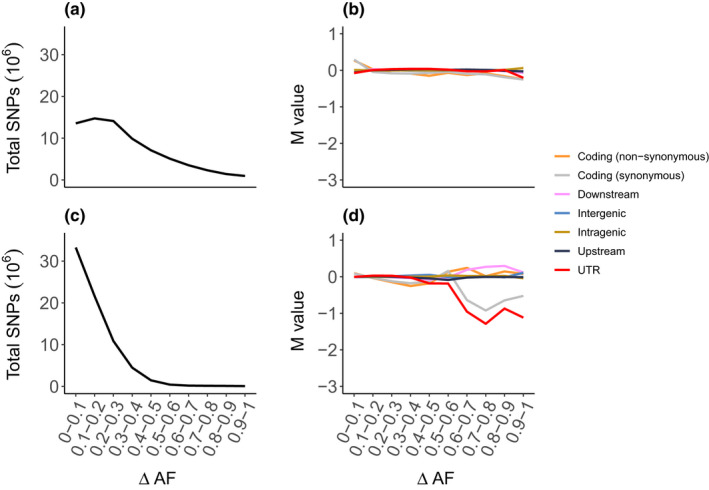
Analysis of difference in allele frequency (*ΔAF*) for different functional categories of SNPs. *ΔAF* calculated between (a, b) introduced populations A and B and (c, d) established populations LB and HV for (a, c) total number of SNPs and (b, d) an enrichment analysis of different functional categories of SNPs. *M*‐values show the relative abundance of SNPs in a given *ΔAF* with a given functional annotation and equal the log2fold change of the observed number of SNPs in a given annotation category for a specific interval of *ΔAF* against the expected SNP count (Table S7). Positive values show that observed frequency is more than expected under neutrality, whereas negative shows that observed frequencies are less than expected. *M*‐values and results from significance testing (*χ* ^2^‐tests) are presented in Table S7

Differences in allele frequencies of the major allele (*ΔAF*) visualized within 5 kb windows show similar genetic relationships as *F*_ST_. *ΔAF* between the introduced populations A and B exceeds *ΔAF* between the established populations LB and HV (Wilcoxon test: *W* = 1.37 × 10^14^, *p* < 2.2 × 10^−16^; Figure 5). For introduced population A and introduced population B, 90% of SNPs exhibit *ΔAF* below 0.6 (Figure 5a), whereas 90% of the allele frequency differences are below 0.2 for the established populations LB and HV (Figure 5c). Although the majority of alleles in the genome segregate at intermediate frequencies in all four groups, there are 8739 windows (out of in total 174,763 windows) that show high *ΔAF* between introduced populations (above 95th percentile of *ΔAF*; *ΔAF* ≥ 0.26). Genetic distances based on these windows mirror the dendrogram: established population LB is alike introduced population B, whereas established population HV is more similar to introduced population A. However, introduced population B is more similar to established population LB than introduced population A is to established population HV (Figure 4b,c).

### 3.4 Functional impact of divergence

Although a few functional categories (i.e., of the types of genetic functions illustrated in Figure 5b,d) are significantly enriched for divergence between introduced populations based on *χ* ^2^‐tests summarized in Table S7, none of their corresponding *M*‐values exceed the cutoff of 0.05, suggesting that the majority of differences between the introduced populations A and B are mainly caused by genetic drift (Figure 5b). Further, of the 319,274 SNPs with differences above the 95th percentile of *ΔAF* between the introduced populations A and B (*ΔAF *> 0.73) and exhibiting a significant difference in allele frequency between introduced populations as tested by Fisher's exact test (significance threshold *p* < 2.5 × 10^−11^), the majority are intragenic (mainly within introns), intergenic, or found upstream of a gene (Table S8). Genetic differences between the introduced populations A and B may primarily be located in functionally less important regions of the genome, and population divergence is mostly a product of drift. Alternatively, functional SNPs have been incorrectly classified due to linkage or are not detected in the present Pool‐seq data.

Similarly, no significant enrichment of any functional category is found when contrasting allele frequencies between the established populations LB and HV (Figure 5d; Table S7).

### 3.5 Adaptive divergence between the introduced populations A and B

Measures of divergence (*F*_ST_) and diversity (*π*) are combined to identify adaptive divergence between the introduced populations A and B. A total of 403 putatively adaptive windows are identified (Figure S4A). Twenty‐one SNPs leading to non‐synonymous changes are found within these windows, of which 20 exhibit significant allele frequency difference between the two introduced populations (Fisher's exact test; *p* < 2.5 × 10^−11^; Table S10). Most of the SNPs are near fixation for alternate alleles in introduced population A and B while intermediate in established populations LB and HV, suggesting hybridization in the new lake system. However, allele frequencies at sites close to fixation in introduced population B are always considerably higher in established population LB than in established population HV, whereas the reverse is true for sites close to fixation in introduced population A (Table S10).

The 20 non‐synonymous SNPs are found within14 genes, marked as candidates for adaptive divergence between introduced populations. Five of these are associated with vertebrate immunology (Table S10), of which three have been described in teleost fish; *RGS5* and *CHD6* in Atlantic salmon (Dettleff et al., 2017; Tacchi et al., 2011) and *SYLT2* in cod (Kleppe et al., 2013). Two additional genes, *CHD23* and *TECTA*, are associated with hearing in zebrafish (*Danio rerio*; Söllner et al., 2004). *CHD23* is associated with a c. 2 Mb region on chromosome 2 containing many fixed SNPs between introduced populations A and B (Figure S4B) that is clearly visible in the Manhattan plot of genome‐wide *F*_ST_ (Figure 3a–d). Yet, another candidate gene, *TELT*, is a component of titin fragments in striated muscle, and expression of this gene is associated with muscle texture in Atlantic salmon (Ørnholt‐Johansson et al., 2017).

### 3.6 Novel selection in the new lake system

We find indications of both forms of selection acting on fish released to a new lake system presently investigated: recent adaption from standing variation through directional selection (Barrett & Schluter, 2008) and relaxed selection (Lahti et al., 2009).

#### 3.6.1 Directional selection

We find 15 5 kb windows possibly shaped by directional selection in the new lake system, as indicated by *ZH*_P_ < −2 in established populations LB and HV and *ZH*_P_ > 0 in both introduced populations A and B. Both established populations LB and HV show greater differentiation to introduced population A than to introduced population B for these windows and while differences in *ΔAF* are low, they are statistically significant (Wilcoxon test established population LB: *p* < 2.5 × 10^−11^, *W* = 2334 and established population HV: *p* < 2.5 × 10^−11^, *W* = 1185; Figure S3). This suggests an advantage of introduced population B alleles over introduced population A in these regions.

Three of the candidate windows for directional selection flank each other on chromosome 7. Fourteen SNPs within this region reflect non‐synonymous changes. The highest *F*_ST_‐values for these non‐synonymous SNPs are found when comparing introduced population A to the three other populations. Ten gene models are predicted (Table S12), of which two regulate metabolism in other salmonids; *LOC106602895* which encodes the protein transcription factor Sox‐19a‐like and is downregulated in food‐deprived Arctic charr (*S. alpinus*; Striberny et al., 2019) and *FOXO1*, a transcription factor involved in metabolic regulation of food intake in Rainbow trout (Conde‐Sieira et al., 2018). An additional gene candidate described in salmonids is *FAXDC2*, which regulates fatty acid synthesis in Atlantic salmon exposed to different experimental diets (Caballero‐Solares et al., 2018). *F*_ST_ between introduced population B and either of the two established populations is generally near zero within these genes.

#### 3.6.2 Relaxed selection

We identify 38 windows putatively shaped by relaxed selection in the new lake system, that is, with *ZH*_P_ < −4 in the introduced populations A and B and *ZH*_P_ > 0 in established populations LB and HV. Chromosome 2 contains a cluster of candidate windows that are associated with a c. 2 Mb region of marked differentiation between introduced populations A and B also found when searching for adaptive differences between the introduced populations based on *F*_ST_ and *π* in the previous section. Most of the SNPs in this region are near fixation for alternate alleles in introduced population A and B while intermediate in established populations LB and HV, suggesting relaxed selection in the new lake system, although hybridization cannot be excluded.

Chromosome 28 is also found to house many neighboring windows putatively shaped by relaxed selection within both established populations LB and HV. Two of these lie within a c. 1 Mb region containing a swarm of SNPs at fixed, or near fixed frequencies. Seven of these are non‐synonymous and encode five genes, of which two (LOC115165611 and LOC115165612) are associated with immunity in salmonids (Zueva et al., 2018; Table S11).

## 4 DISCUSSION

Genome‐wide intraspecific variability is monitored over contemporary time following an artificial one‐time‐only introduction of brown trout to a novel lake system previously void of the species. The results suggest that both introduced populations A and B have contributed to fish established in the wild in downstream lakes Lilla Bävervattnet (LB) and Haravattnet (HV). This contention is supported by genome‐wide divergence and diversity in the four groups of fish. The genome‐wide divergence between introduced populations A and B exceeds the divergence between fish established in the new lake system, as indicated by *F*_ST_ (Figure 3) and a dendrogram of genetic distances (Figure 4). Within‐population variability has, however, increased in the new lake system: genome‐wide variation within the established populations LB and HV exceeds that of introduced populations A and B (see Table S5 for statistical tests). Diversity is lowest in introduced population B, whereas highest in the established population HV (Wilcoxon test: *W =* 452, *p* < 2.2 × 10^−16^; Table S5). Generally, presently estimated population metrics appear low in comparison to observations from other wild salmonid populations (Leitwein et al., 2016; Willoughby et al., 2018). However, our own studies of natural brown trout in nearby mountain lake systems (ecologically similar to the system studied here), using a variety of genetic markers, indicate that the diversity levels of the established populations LB and HV are among the highest observed (Andersson et al., 2017; Kurland et al., 2019; Palm et al., 2003; Palmé et al., 2013; Saha et al., 2022). It is interesting to note that even though the established population HV is located several kilometers away from the release site, genetic diversity is highest here.

Hybridization between introduced populations A and B is corroborated by a limited SNP panel (comprising 96 SNPs) employed to study fish populating this general mountain area (A. Andersson, L. Laikre, N. Ryman, unpublished), as well as by Palm and Ryman (1999) in their study of fish following the introduction of populations A and B using allozymes. Palm and Ryman (1999) found hybrids between fish from the two source populations among the first‐generation offspring produced in the new lake system. However, there were fewer hybrids than expected under random mating, suggesting preferential mating within fish from the two source populations (their data do not support reduced survival of hybrids, rather they report weak indications of better survival in hybrids). Perhaps, hybridization was inevitable in the new lake system. Alternatively, hybridization may have been beneficial, as has been observed in cichlid fish (Meier et al., 2019) and yeast (Zhang et al., 2020).

Interestingly, we find that genetic contributions from introduced populations A and B to established populations LB and HV are unequal. Introduced population B, which is non‐migratory in its original habitat (Lake Fälpfjälltjärnarna), has contributed more to established population LB in the lake closest to the release sites. In contrast, introduced population A, which is migratory in its natal environment (Lake Kallsjön), has contributed more to the established population HV furthest downstream in the system. This trend is observed across the genome (Figures 3 and 4), but also for SNPs exhibiting the most extreme divergence between introduced populations (Figure 4b,c). Additionally, for candidates of directional selection in the new lake system, allele frequencies within established populations LB and HV are more similar to introduced population A than B (Figure S3, Table S12). These observations suggest that alleles from the small, close‐by lakes that introduced population B originates from (Lake Fälpfjälltjärnarna) —with similar ecological conditions as the lakes sampled here—have remained close to the area of release, whereas alleles from introduced population A (Lake Kallsjön), that is migratory in its natal habitat, have spread further away from the site of release.

The unequal contribution of introduced populations to lakes in the new system is mirrored in the 96 SNP panel (A. Andersson, L. Laikre, N. Ryman, unpublished data). Further, Palm and Ryman (1999) studied fish in Lake Stora Bävervattnet not included in the present study as well as the presently included Lake Lilla Bävervattnet (Figure 1). They found that introduced population B genes are more common in Lake Bävervattnet as compared to Lake Stora Bävervattnet. In the present study, we sample fish from Lake Haravattnet, which is even further downstream in the system, and find that in this lake too, introduced population A genes are more common. Introduced population A has seemingly maintained a larger geographic spread, whereas introduced population B dominates the lake nearest to the release site, which was attributed by Palm and Ryman (1999) to the successful reproduction of this introduced population during the first few years. Our finding, contested by the 96 SNPs and Palm and Ryman (1999), implies that the two introduced populations A and B have been successful in the new lake system by employing divergent strategies, in addition to extensive hybridization having occurred.

### 4.1 Adaptive divergence between introduced populations A and B

The introduced populations A and B were initially chosen to represent different body sizes and life history adaptations, for example, growth rate, and migratory and reproductive behaviors (Appendix S1; Palm & Ryman, 1999). In their common garden experiment on which the present study is founded, Palm and Ryman (1999) confirm a genetic basis for the most distinctive characteristics of source populations. Presently, genes putatively associated with an adaptative divergence between introduced populations A and B are identified to have functions possibly related to immunity, hearing, and muscle texture. Local selection for genes associated with immunology exists over small geographic scales in other salmon populations and is therefore expected (Kjærner‐Semb et al., 2016, 2021; Pritchard et al., 2018; Zueva et al., 2018). However, traits such as those characterizing introduced populations A and B are complex. Some life history traits, for example, migratory and reproductive behavior, are influenced by intrinsic traits, for example, metabolism (Eldøy et al., 2021). These behaviors are, in turn, affected by environmental factors that may vary within populations over time and are governed by complex genetic architectures (Debes et al., 2021; Näslund et al., 2018). Further study on the dynamics of phenotypes and underlying genes is warranted.

### 4.2 Novel selection in the new lake system

We identified regions with low heterozygosity scores in established populations LB and HV compared to the introduced populations A and B, suggesting direction selection in the new environments. For these regions, we find that both established populations show greater differentiation to introduced population A than to introduced population B (Wilcoxon test established population LB: *W* = 1462, *p* < 2.5 × 10^−11^ and established population LB: *W* = 1100, *p* < 2.5 × 10^−11^; Figure S3).

Of the regions putatively under directional selection in the new lake system, we found three genes on chromosome 7 *(LOC106602895*, *FOXO1*, and *LARP1*) of marked differentiation between introduced population A and both established populations. These genes are all associated with metabolism, and *F*_ST_ between the two introduced populations A and B is high. This indicates that the metabolic requirements, for example, nutrient availability within the Lakes Lilla Bävervattnet and Haravattnet are more similar to those of the small lakes from which introduced population B originates than those of the larger lake that introduced population A is from. It is also striking to find genes related to metabolism since such intrinsic traits may underly other behaviors, for example, migratory and reproductive (Eldøy et al., 2021), where the two introduced populations A and B differ.

### 4.3 Limitations

Monitoring intraspecific diversity over a few generations poses difficulties in estimating allele frequency shifts since it is unlikely that the established populations LB and HV are in equilibrium with respect to linkage disequilibrium (LD), gene flow, and drift (Hössjer & Ryman, 2014). This is a common problem in many situations of contemporary monitoring of human‐induced effects on microevolutionary patterns. First, with regard to the linkage, Leitwein et al. (2016) report a significant, positive, correlation between nucleotide diversity and recombination rate across the brown trout genome, making it likely that selection is limiting variation at linked neutral sites. We acknowledge that linkage disequilibrium may confound our description of functional divergence between introduced populations and search for adaptive loci.

Second, fish in Lakes LB and HV are descendants of the introduced fish and each individual in these lakes constitutes a mosaic of parental alleles. Combinations of parental alleles will shuffle over generations, in part due to drift, LD, and recombination (Jacobs et al., 2020). Contrasting descendant fish to parents from five generations ago may thus create artificial signals of selection and elevated shifts in allele frequency underlying diversity estimates (Jorde & Ryman, 1995; Palm et al., 2003). Additionally, while a sample size of 50 is sufficient to detect changes in allele frequency in Pool‐seq data, larger sample sizes may be required to detect very subtle shifts (Kofler, Orozco‐terWengel, et al., 2011; Schlötterer et al., 2014).

Third, correcting for drift is difficult when population histories are unknown. Our search for adaptive variation between introduced populations A and B focused on areas of the genome showing elevated divergence between introduced populations. In order to avoid confounding selection with other evolutionary forces, for example, drift, we combined measures of divergence with diversity over 5 kb windows (Carneiro et al., 2014; Kjærner‐Semb et al., 2016). This approach poses additional problems. First, divergence is variable across the genome and local inflations in differentiation may be due to reduced diversity in regions shaped by recombination (e.g., surrounding centromeres) or increased background selection (e.g., in regions with high gene density; Jacobs et al., 2020). This can create false identification of selection. However, given so few generations, recombination is most likely not prominent in the current study.

Fourth, structural variation including copy number variation is prevalent among polyploid salmonids (Brenna‐Hansen et al., 2012; Lien et al., 2016). This may bias inferences of selection yet is not a primary source of concern for the current candidates of selection in the new environment as they do not show elevated read depth in comparison to genome‐wide levels, nor are they represented by few individuals (paired *t*‐test: *p* > .05; Table S9). Candidate SNPs for adaptive divergence between introduced populations A and B exhibit higher coverage than a random sample of equal size (average read depth is 74 and 63, respectively, paired *t*‐test: df = 21, *t* = 2.73, *p* = .01). However, the magnitude of this difference, estimated as fold change, is near zero (*M* = 0.24; Table S9). Since we generally apply stringent read depth filters in order to avoid false positives, a more pressing limitation involves overlooking structural variation of significance for population viability (Bertolotti et al., 2020; Wellband et al., 2019)—for which further study is warranted.

In an approach to identify novel selection in the new lake system and to avoid regions shaped by drift, we focus on regions containing many fixed loci in contrasting population pairs (introduced populations A and B compared to established populations LB and HV). We use stringent cutoffs in order to represent the extremes of the distribution (Rubin et al., 2010). However, without correcting for multiple testing, there is a risk of false identifications of selective loci.

There are many limitations of this study that warrant follow‐up research, and we are planning for such work. Nevertheless, attempting to monitor contemporary genomic changes also in non‐equilibrium situations and for species with complex genomes and population structures allowing extensive genetic drift is highly warranted in the light of the ongoing biodiversity crisis. We hope that this study will provide initial insights that can develop our understanding of microevolutionary genomics in the era of Anthropocene.

## AUTHOR CONTRIBUTIONS

**Sara Kurland:** Conceptualization (supporting); data curation (lead); formal analysis (lead); methodology (lead); resources (supporting); visualization (lead); writing – original draft (lead). **Nima Rafati:** Formal analysis (supporting); methodology (supporting); supervision (supporting); writing – review and editing (equal). **Nils Ryman:** Conceptualization (lead); formal analysis (supporting); supervision (equal); writing – review and editing (equal). **Linda Laikre:** Conceptualization (lead); formal analysis (supporting); funding acquisition (lead); project administration (lead); resources (lead); supervision (equal); writing – review and editing (equal).

## CONFLICT OF INTEREST

The authors declare no conflict of interest.

## Supporting information


Supplementary material

## Data Availability

Illumina raw sequences from this study have been deposited in the European Nucleotide Archive (ENA) at EMBL‐EBI under project accession number PRJEB48212 and study accession number ERP132551.
