# Supplementary material for: Genomic dynamics of brown trout populations released to a novel environment
Source: Ecol Evol. 2022 Jul 3;12(7):e9050. doi: 10.1002/ece3.9050 (PMC9251865; doi:10.1002/ece3.9050)
Supplement: Supplementary file 1 — Supplementary material [file ECE3-12-e9050-s001.pdf]

Supporting Information for:

Genomic dynamics of brown trout populations released to a novel environment

Sara Kurland, Nima Rafati, Nils Ryman, Linda Laikre

Table of Contents:

**Appendix S1**	Detailed material and methods	Page 2
**Table S1**	Mapping statistics	Page 6
**Table S2**	Number of variants and windows used for diversity metrics	Page 6
**Table S3**	Pooled heterozygosity score (H_P) for each population pool	Page 6
**Table S4**	Statistical tests for amount H_P between pairwise pools	Page 7
**Table S5**	Statistical tests for π between pairwise pools	Page 7
**Table S6**	Statistical tests for Tajima's D between pairwise pools	Page 7
**Table S7**	Distributions of SNP counts in the different delta allele frequency bins for different functional impacts.	Page 8-9
**Table S8**	Functional categories of SNPs fixed between introduced populations	Page 10
**Table S9**	Read depth for non-synonymous SNPs identified as candidates for adaptive divergence between introduced populations A and B, and candidates for novel selection in the new lake system	Page 10
**Table S10**	Genes of adaptive importance for introduced populations	Page 11-12
**Table S11**	Genes putatively shaped by relaxed selection in the new environments on chromosome 28	Page 13
**Table S12**	Genes putatively under directional selection in the new environments on chromosome 7	Page 14-15
**Table S13**	Glossary for some of the terminology used	Page 17
**Figure S1**	Genome wide heterozygosity per pool (H_P) in 5 kb windows.	Page 18
**Figure S2**	Genome-wide nucleotide diversity (π) and Tajima's D (T_D)	Page 19
**Figure S3**	Boxplots of difference in allele frequency (ΔAF) between each of the introduced populations A and B and established populations LB and HV for putatively selective regions	Page 20
**Figure S4**	Potentially adaptive differences between the two introduced populations A and B	Page 21

Appendix S1: Detailed material and methods

Study system

The introduced populations A and B exhibited significant allele frequency differences at multiple allozyme loci, particularly at *AGP-2*, where the population from Lake Kallsjön was close to fixation for one of the alleles at this locus, while in Lakes Fälpfjälltjärnarna two alleles occurred at about equal frequencies (Palm & Ryman 1999). Spawners from each population were chosen to be homozygous for different alleles at this locus, thereby enabling discrepancy of lineages after release to the same environment (Palm & Ryman 1999). Brown trout established rapidly in the lakes Bävervattnen area after the release and have been sampled continuously with tissues stored in -80° C freezers. The first documented reproduction occurred only a few years after the release (Palm & Ryman 1999) and ten years later both lakes downstream of the release site harbored strong populations from which sampling was easy. During the period 2003-2007 several lakes below the release site were sampled and brown trout establishment confirmed in all of those lakes (Figure 1; unpublished data).

Table. Sampling information for the material used in this study (cf. Figure 1).

Lake	Geographic location (coordinates in WGS84 dec) N	E	Area (km$^{2)}$
Lilla Bävervattnet (established population LB)	64.048928	14.633335	0.132
Haravattnet (established population HV)	64.027934	14.715422	0.121
Kallsjön (source population introduced population A)	63.6167	13.0000	158
Fälpfjälltjärn p 645, northern (source population introduced population B)	64.181465	14.739145	0.1281
Fälpfjälltjärn p 671, southern (source population introduced populations B)	64.173004	14.737597	0.0929

DNA extraction

Genomic DNA was extracted and eluted in 100 µl elution buffer. DNA fragmentation was assessed on 2% agarose gels stained with GelRed and absorbance at 260/280. High molecular weight DNA from each individual was quantified using fluorometry (Qubit; Thermo Scientific) and combined at equal concentrations for each population to achieve 3 µg pooled genomic DNA in a volume within the range of 65–120 µl.

Library construction and sequencing

PCR-free paired-end libraries had an average insert size of 350 bp, sequencing used read length 150 bp and was performed by NGI across 3 (introduced populations A and B) or 4 (established populations LB and HV) lanes per pool.

Mapping and variant calling

The quality of sequenced reads from each pool were assessed using FastQC v.0.11.5 (Leggett et al. 2013) and results from different pools jointly evaluated using MultiQC v.1.5 (Ewels et al. 2016). Quality assessments of bam files were obtained from SAMtools flagstat and Qualimap v.2.2.1 (García-Alcalde et al. 2012), and summarized in MultiQC v.1.5 (Ewels et al. 2016). Variant calling was conducted in SAMtools using minimum base and mapping quality scores of 20 and the parameter "base alignment quality" (BAQ; "-B") to reduce false SNPs caused by misalignments, resulting in one mpileup file for all four pools. The mpileup file was then converted to synchronized format using the 'mpileup2sync.jar' script of PoPoolation2 v.1.201 and used for downstream analyses of allele frequencies and F_{ST} in PoPoolation2 v.1.201. The mpileup file was also split for estimation of within-population metrics in PoPoolation v.2.2

(Kofler et al. 2011a), which requires pool-based pileup files. Estimates of within-population metrics from Pool-seq data are sensitive to sequencing errors and variation in coverage (Kofler et al. 2011a). The mpileups were therefore subsampled without replacement to uniform depths (20-150X) using the 'subsample-pileup.pl' script implemented in PoPoolation v.2.2 (Kofler et al. 2011a). Depth thresholds were chosen based on the mode of the read depth histogram for each pool (using a minimum depth of 0.5xmode and a maximum depth of mode+0.5xmode for subsampling; see Kurland et al. 2019).

VCF files were created from bam files using BCFools v.1.10 (Li et al. 2009) by calling and genotyping raw variants in the software's mpileup and call algorithms, using default settings. Variants were compared with those called in the estimation of allele frequencies in Popoolation2 v1.201 (Kofler et al. 2011b), only keeping sites found in both files. These were then controlled for mapping quality, number of high-quality bases, read positional bias, base quality bias, and mapping quality versus strand bias, before filtering for mapping quality 100. The final set (7.4×10^6 variants) were annotated in SnpEff v.5.0 (Cingolani et al. 2012).

Patterns of genomic variation and divergence
Nucleotide diversity (π; Tajima, 1983) and Tajima's D (T_D; Tajima, 1989) were estimated using the 'variance-sliding.pl' script of PoPoolation v.2.2 (Kofler et al. 2011a) in 5 kilo base pair (kb) non-overlapping windows, with a minor allele count of 2 for a SNP to be called and applying the same depth thresholds as for the subsampling (20-150X). Windows were retained for subsequent analysis if at least 80% of the window was covered with data within these depth thresholds.

We primarily used the default F_{ST} (Nei 1973) but also computed the alternative option in PoPoolation2 which is the approach of Karlsson et al. (2007) since these two approaches have different merits (Saha et al. 2021, their Supporting Appendix S4). A minor allele count of 3 was applied for SNP calling. Windows were only retained if the fraction of the number of sites within a window covered with data exceeded 80%. Genetic relationships among populations were examined by creating a dendrogram in TreeMix (Pickrell & Pritchard 2012), which constructs a maximum likelihood phylogeny based on the genomic data and compares the covariance structure calculated for the estimated dendrogram to the observed covariance between populations. TreeMix was run with default settings and the results visualized in MEGA X (Kumar et al. 2018).

Genomic distribution of SNPs
SnpEff v.5.0 (Cingolani et al. 2012) was used to annotate the genomic distribution of variants and to classify them into functional elements (non-synonymous, synonymous, untranslated region (UTR), 5 kb upstream, 5 kb downstream, intragenic, and intergenic). Functional elements from less confidant annotations, e.g. missing start and stop codons from the transcript (Barrio et al. 2016), were omitted and some functional elements were combined in order to avoid too small classes at high ΔAF (e.g. 3' UTR and 5' UTR combined to functional class "UTR"). For each of these functional categories, the allele frequency differences between introduced populations were sorted into bins (10 equally large bins of ΔAF=0-0.1, ΔAF=0.1-0.2 etc.). Log2 fold change was retrieved by comparing the observed and expected number of SNPs per category and bin. The expected number of SNPs in each category and bin was estimated as p(category) x n(bin), where p(category) equals the proportion of a given SNP category in the full genome and n(bin) the number of SNPs in an allele frequency bin. Statistical significances of deviations of observed from expected SNP counts were tested with standard chi-square tests of independence.

Screening for indications of selection in the new environment

We screened for signatures of selection in the new environments by considering local reductions of heterozygosity score within each pool (H_P) relative to its pool-based genome-wide average (cf. Rubin et al. 2010; Kardos et al. 2015; Kjærner-Semb et al. 2016; Willoughby et al. 2018). Normalized H_P (ZH_P) was used in this approach $ZH_P = (H_P - \mu H_P)/ \sigma H_P)$ as calculated per population pool (following Rubin et al. 2010).

In order to identify putatively selected genes in the new environment, we selected windows (5 kb) of particularly low ZH_P out of the windows with ZH_p <0 in descendant populations (below 5th percentile of ZH_P in LB and HV, respectively). Precedence was given to the windows with lowest levels of ZH_P in both descendant populations and regions with multiple adjacent windows. Patterns of population divergence (F_{ST}) surrounding these windows was then studied to corroborate assumptions of selection. The same approach was given to windows of markedly low diversity within introduced populations. The functional annotation of SNPs within windows of low H_P in introduced populations and established populations respectively were extracted from the VCF annotated in SnpEff v.5.0 (Cingolani et al. 2012).

References for Appendix S1

Carneiro, M., Rubin, C. J., Di Palma, F., Albert, F. W., Alföldi, J., Barrio, A. M., ... & Younis, S. (2014). Rabbit genome analysis reveals a polygenic basis for phenotypic change during domestication. *Science*, *345*(6200), 1074–1079.

Cingolani, P., Platts, A., Wang, L. L., Coon, M., Nguyen, T., Wang, L., ... & Ruden, D. M. (2012). A program for annotating and predicting the effects of single nucleotide polymorphisms, SnpEff: SNPs in the genome of Drosophila melanogaster strain w1118; iso-2; iso-3. *Fly, 6*(2), 80-92.

Ewels, P., Magnusson, M., Lundin, S., & Käller, M. (2016). MultiQC: summarize analysis results for multiple tools and samples in a single report. Bioinformatics, *32*(19), 3047-3048.

García-Alcalde, F., Okonechnikov, K., Carbonell, J., Cruz, L. M., Götz, S., Tarazona, S., . . . Conesa, A. (2012). Qualimap: evaluating next-generation sequencing alignment data. *Bioinformatics, 28*(20), 2678-2679. doi:10.1093/bioinformatics/bts503

Karlsson, E.K., Baranowska, I., Wade, C. M., Salmon Hillbertz, N. H. C., Zody, M. C., anderson N., Biagi, T. M., Patterson, N., Rosengren Pielberg, G., Kulbokas III, E. J., Comstock, K. E., Keller, E. T., Mesirov, J. P., von Euler, H., Kämpe, O., Hedhammar, Å., Lander, E. S., Andersson, G., Andersson, L., & Lindblad-Toh, K. (2007). Efficient mapping of mendelian traits in dogs through genome-wide association. Nature Genetics *39*(11), 1321-1328.

Kardos M, Luikart G, Bunch R, Dewey S, Edwards W, McWilliam S, Stephenson J, Allendorf FW, Hogg JT, Kijas J. 2015. Whole-genome resequencing uncovers molecular signatures of natural and sexual selection in wild bighorn sheep. Molecular Ecology 24:5616–5632.

Kjærner-Semb E et al. 2016. Atlantic salmon populations reveal adaptive divergence of immune related genes - a duplicated genome under selection. BMC genomics 17:610. BMC Genomics.Kofler, R., Orozco-terWengel, P., De Maio, N., Pandey, R. V., Nolte, V., Futschik, A., . . . Schlötterer, C. (2011). PoPoolation: a toolbox for population genetic analysis of next generation sequencing data from pooled individuals. PLOS ONE, 6(1), e15925. doi:10.1371/journal.pone.0015925

Kofler, R., Pandey, R. V., & Schlötterer, C. (2011). PoPoolation2: identifying differentiation between populations using sequencing of pooled DNA samples (Pool-Seq). *Bioinformatics, 27*(24), 3435-3436. doi:10.1093/bioinformatics/btr589

Kumar, S., Stecher, G., Li, M., Knyaz, C., & Tamura, K. (2018). MEGA X: molecular evolutionary genetics analysis across computing platforms. Molecular Biology and Evolution, *35*(6), 1547-1549. doi:10.1093/molbev/msy096

Kurland, S., Wheat, C. W., de la Paz Celorio Mancera, M., Kutschera, V. E., Hill, J., Andersson, A., … Laikre, L. (2019). Exploring a Pool-seq-only approach for gaining population

genomic insights in nonmodel species. *Ecology and Evolution*, *9*(19). https://doi.org/10.1002/ece3.5646
Lamichhaney, S., Han, F., Webster, M. T., Grant, B. R., Grant, P. R., & Andersson, L. (2020). Female-biased gene flow between two species of Darwin's finches. *Nature Ecology & Evolution*, *4*(7), 979-986.
Leggett, R. M., Ramirez-Gonzalez, R. H., Clavijo, B. J., Waite, D., & Davey, R. P. (2013). Sequencing quality assessment tools to enable data-driven informatics for high throughput genomics. *Frontiers in Genetics, 4*, (288), 1-5. doi: 10.3389/fgene.2013.00288
Li, H. (2013). Aligning sequence reads, clone sequences and assembly contigs with BWA-MEM (Publication no. arXiv:1303.3997v2 [q-bio.GN]).
Li, H., & Durbin, R. (2009). Fast and accurate short read alignment with Burrows–Wheeler transform. *Bioinformatics*, *25*(14), 1754-1760. doi:10.1093/bioinformatics/btp324
Li, H., Handsaker, B., Wysoker, A., Fennell, T., Ruan, J., Homer, N., . . . Subgroup, G. P. D. P. (2009). The sequence alignment/map format and SAMtools. *Bioinformatics*, *25*(16), 2078-2079. doi:10.1093/bioinformatics/btp352
Nei, M. (1973). Analysis of gene diversity in subdivided populations. *Proceedings of the National Academy of Sciences*, *70*(12), 3321-3323. doi:10.1073/pnas.70.12.332
Palm, S., & Ryman, N. (1999). Genetic basis of phenotypic differences between transplanted stocks of brown trout. *Ecology of Freshwater Fish*, *8*(3), 169–180. https://doi.org/10.1111/j.1600-0633.1999.tb00068.x
Palmé, A., Wennerström, L., Guban, P., Ryman, N., & Laikre, L. (2012). Compromising Baltic salmon genetic diversity – conservation genetic risks associated with compensatory releases of salmon in the Baltic Sea. Swedish Agency of Marine and Water Management Report 2012:18. https://www.havochvatten.se/download/18.13780b7613b461ffa9e1a83/1355996777952/rapport-2012-18-compromising-baltic-salmon-english.pdf
Pickrell, J. K., & Pritchard, J. K. (2012). Inference of population splits and mixtures from genome-wide allele frequency data. *PLOS Genetics*, *8*(11), e1002967. doi:10.1371/journal.pgen.1002967
Rubin, C. J., Zody, M. C., Eriksson, J., Meadows, J. R., Sherwood, E., Webster, M. T., ... & Andersson, L. (2010). Whole-genome resequencing reveals loci under selection during chicken domestication. *Nature*, *464*(7288), 587-591.
Ryman, N., Öhman, R., Ståhl, G., Nilsson, A,, Lagercrantz, U., & Herlitz, A. 1986. Genetic variation in salmonid fishes - a rapidly declining natural resource: information on the scientific work in the Lakes Bävervattnen area in Jämtland County, central Sweden. Resta Grafiska AB, Stockholm (in Swedish).
Saha, A., Andersson, A., Kurland, S., Keehnen, N. L., Kutschera, V. E., Hössjer, O., ... & Laikre, L. (2021). Whole-genome resequencing confirms reproductive isolation between sympatric demes of brown trout (*Salmo trutta*) detected with allozymes. *Molecular Ecology*.
Ståhl, G. & Ryman, N. 1982. Simple Mendelian inheritance at a locus coding for alpha-glycerophosphate
dehydrogenase in brown trout (Salmo trutta). *Hereditas 96*:313-315.
Tajima, F. (1983). Evolutionary relationship of DNA sequences in finite populations. *Genetics, 105*(2), 437-460.
Tajima, F. (1989). Statistical method for testing the neutral mutation hypothesis by DNA polymorphism. *Genetics, 123*(3), 585-595.
Willoughby JR, Harder AM, Tennessen JA, Scribner KT, Christie MR. 2018. Rapid genetic adaptation to a novel environment despite a genome-wide reduction in genetic diversity. Molecular Ecology **27**:4041–4051.

Table S1. BAM file statistics from QualiMap for Pool-seq data from 4 populations pools mapped to the *S. trutta* assembly (size 2.4 Gb), using bwa mem and default settings. Results are presented for properly paired-end reads with minimum base quality 20. Average coverage and its standard deviation (*SD*), and mean mapping quality is also included. Down-stream analyses were additionally limited to reads with read depth 20-150X.

Statistic	Introduced population A	Introduced population B	Established population LB	Established population HV
Number of sequenced bases (x 10^9)	194	209	166	192
Number of reads mapped as pairs (x 10^9)	1.45	1.54	1.42	1.22
Percentage of reads mapped as pairs (%)	92.5	93.0	93.2	93.4
Average depth of coverage	82	88	81	70
Mean mapping quality	32.9	33.1	32.9	33.1
General error rate (%)	0.61	0.61	0.59	0.61

Table S2. Summary of number of variants and windows (5 kb in size) supporting each diversity metric. The mpileup file used $1.8x10^9$ variant loci of which only biallelic SNPs, mapped to chromosomes (no orphans), and within coverage 20-150X were used for estimating population genomic parameters. Minimum coverage of 80% (min. cov. 0.8) implies whether windows were retained for subsequent analysis if at least 80% of the window was covered with data within these depth thresholds (marked "Yes"), otherwise (marked "-") all windows were used. Allele frequency was only estimated per variant site and not within windows.

Diversity measure	No. SNPs	No. 5 kb windows	Read depth	Min. cov. 0.8
Allele frequency and pooled heterozygosity score (H_P)	10,651,540	174,763	20-150X	-
Nucleotide diversity and Tajima's D	5,740,787	331,179	20-150X	Yes
F_{ST}	11,007,131	329,853	20-150X	Yes

Table S3. Genome-wide pooled heterozygosity score for each of the population pools (H_P; Rubin et al. 2010) along with 95% confidence intervals estimated within 5 kb windows.

Population	Mean H_P	95% CI
Introduced population A	0.180	0.1798-0.1806
Introduced population B	0.162	0.1613-0.1621
Established population LB	0.189	0.1885-0.1892
Established population HV	0.195	0.1943-0.1951

Table S4. Pairwise comparisons of genome wide heterozygosity per pool (H_P) in Wilcoxon rank sum test. Significance threshold $p<2.5\text{x}10^{-11}$ (α=0.05 corrected for genome size of 2.4 Gb).

Comparison	W (x10^{12})	p (W)
Introduced population A: Introduced population B	117	$<2.2 \times 10^{-16}$
Introduced population A: Established population LB	104	$<2.2 \times 10^{-16}$
Introduced population A: Established population HV	104	$<2.2 \times 10^{-16}$
Introduced population B: Established population LB	97	$<2.2 \times 10^{-16}$
Introduced population B: Established population HV	97	$<2.2 \times 10^{-16}$
Established population LB: Established population HV	110	$<2.2 \times 10^{-16}$

Table S5. Pairwise comparisons of genome wide nucleotide diversity (π) within population pools in Wilcoxon rank sum test. Significance threshold $p<2.5\text{x}10^{-11}$ (corresponding to α=0.05 corrected for multiple testing across a genome size of 2 Gb).

Comparison	W	p (W)
Introduced population A: Introduced population B	606	$<2.2 \times 10^{-16}$
Introduced population A: Established population LB	531	$<2.2 \times 10^{-16}$
Introduced population A: Established population HV	510	$<2.2 \times 10^{-16}$
Introduced population B: Established population LB	473	$<2.2 \times 10^{-16}$
Introduced population B: Established population HV	452	$<2.2 \times 10^{-16}$
Established population LB: Established population HV	526	$<2.2 \times 10^{-16}$

Table S6. Pairwise comparisons of genome wide Tajima's D (T_D) within population pools in Wilcoxon rank sum test. Significance threshold $p<2.5\text{x}10^{-11}$ (corresponding to α=0.05 corrected for multiple testing across a genome size of 2 Gb).

Comparison	W (x10^{9})	p (W)
Introduced population A: Introduced population B	62	$<2.2 \times 10^{-16}$
Introduced population A: Established population LB	61	$<2.2 \times 10^{-16}$
Introduced population A: Established population HV	57	$<2.2 \times 10^{-16}$
Introduced population B: Established population LB	54	$<2.2 \times 10^{-16}$
Introduced population B: Established population HV	50	$<2.2 \times 10^{-16}$
Established population LB: Established population HV	59	$<2.2 \times 10^{-16}$

Table S7. Distributions of SNP counts in the delta allele frequency (*ΔAF*) bins for different functional categories (non-synonymous and synonymous coding sequences, untranslated region (UTR), 5 kb upstream, 5 kb downstream, intragenic, and intergenic). Binned *ΔAF* are made for the contrast between introduced populations A and B and established populations LB and HV, respectively. Observed and expected number of SNPs of each category and bin were used to estimate *M*-values of log2fold change in observed vs. expected SNP count. Statistical significance of deviations from expected values are tested with stander χ^2-analysis (df=1). P-values in bold indicate those below significance threshold $p<2.5\times10^{-11}$ (α=0.05 corrected for multiple testing across a genome size of 2 Gb).

		***ΔAF* between introduced populations A and B**				***ΔAF* between established populations LB and HV**			
**ΔAF bin**	**Functional category**	**Observed**	**Expected**	***M***	***p***	**Observed**	**Expected**	***M***	***p***
0-0.1	Coding (non-synonymous)	17566	14584.07	0.26839	**1.2999E-134**	38373	35799.8	0.10014	**3.9991E-42**
	Coding (synonymous)	20775	16955.91	0.29306	**4.4063E-189**	44287	41621.9	0.08954	**5.3513E-39**
	Downstream	58130	60302.27	-0.0529	**9.07246E-19**	148582	148025	0.00542	0.1476925
	Intergenic	428279	426427	0.00625	0.004567152	1041704	1046757	-0.007	7.8582E-07
	Intragenic	603032	599013.1	0.00965	2.07255E-07	1471678	1470407	0.00125	0.2945658
	Upstream	198812	207777.4	-0.0636	**4.02371E-86**	508312	510035	-0.0049	0.0158696
	UTR	28174	29708.34	-0.0765	**5.49073E-19**	72635	72925.5	-0.0058	0.28197841
0.1-0.2	Coding (non-synonymous)	16156	15857.34	0.02692	0.01770667	22702	23277.4	-0.0361	0.00016242
	Coding (synonymous)	17850	18436.26	-0.0466	1.5768E-05	26487	27063	-0.031	0.00046261
	Downstream	66930	65567	0.02968	1.0209E-07	96750	96247.4	0.00751	0.10522883
	Intergenic	464348	463656.5	0.00215	0.309866565	677267	680613	-0.0071	5.0016E-05
	Intragenic	648940	651310.3	-0.0053	0.00331336	955524	956075	-0.0008	0.57343218
	Upstream	226344	225917.5	0.00272	0.369569743	335157	331630	0.01526	9.0805E-10
	UTR	32479	32302.05	0.00788	0.32483396	48435	47417	0.03065	2.9377E-06
0.2-0.3	Coding (non-synonymous)	14339	15185.22	-0.0827	**6.55283E-12**	10563	11698.3	-0.1473	**8.9617E-26**
	Coding (synonymous)	16672	17654.82	-0.0826	**1.3951E-13**	12499	13600.8	-0.1219	**3.4676E-21**
	Downstream	63155	62787.9	0.00841	0.142913928	47831	48370.1	-0.0162	0.01423404
	Intergenic	445054	444004.2	0.00341	0.115129984	345266	342049	0.01351	3.7822E-08
	Intragenic	621407	623704.1	-0.0053	0.003629483	479153	480485	-0.004	0.05465537
	Upstream	218363	216341.9	0.01342	1.39051E-05	167088	166664	0.00367	0.29899455
	UTR	31621	30932.9	0.03174	9.13968E-05	24297	23829.9	0.02801	0.0024782
0.3-0.4	Coding (non-synonymous)	9983	10623.07	-0.0897	5.29387E-10	4048	4826.22	-0.2537	**3.9849E-29**
	Coding (synonymous)	11610	12350.72	-0.0892	2.6445E-11	4957	5611.11	-0.1788	**2.4953E-18**
	Downstream	44163	43924.3	0.00782	0.254735099	19420	19955.5	-0.0392	0.00015035
	Intergenic	308559	310610.4	-0.0096	0.000232551	144499	141115	0.03419	**2.0873E-19**
	Intragenic	436016	436322.4	-0.001	0.64271699	198041	198228	-0.0014	0.67497059
	Upstream	154242	151345.5	0.02735	**9.66228E-14**	67664	68758.5	-0.0231	2.994E-05
	UTR	22243	21639.62	0.03968	4.10077E-05	9696	9831.2	-0.02	0.17271952
0.4-0.5	Coding (non-synonymous)	6881	7633.92	-0.1498	**6.8506E-18**	1372	1555.96	-0.1815	3.1064E-06
	Coding (synonymous)	8455	8875.441	-0.07	8.08852E-06	1659	1809.01	-0.1249	0.00042033
	Downstream	31941	31564.76	0.01709	0.034201739	6089	6433.6	-0.0794	1.7377E-05
	Intergenic	222073	223210	-0.0074	0.016104654	47131	45495.1	0.05096	**1.7262E-14**
	Intragenic	312630	313548.8	-0.0042	0.100819666	64017	63908.2	0.00245	0.66683393

	Upstream	111185	108759.5	0.03182	**1.91203E-13**	21476	22167.6	-0.0457	3.4012E-06
	UTR	15978	15550.6	0.03912	0.000609514	2795	3169.56	-0.1814	2.8718E-11
	Coding (non-synonymous)	5229	5483.656	-0.0686	0.000584096	497	452.388	0.13568	0.03595226
	Coding (synonymous)	6191	6375.474	-0.0424	0.020868447	587	525.961	0.1584	0.00777864
	Downstream	22826	22673.84	0.00965	0.312253969	1843	1870.54	-0.0214	0.52433654
0.5-0.6	Intergenic	159730	160337.9	-0.0055	0.128986573	13153	13227.5	-0.0081	0.51724922
	Intragenic	225268	225230.8	0.00024	0.937488843	19066	18581	0.03718	0.00037357
	Upstream	78839	78124.94	0.01313	0.010627725	6067	6445.12	-0.0872	2.4788E-06
	UTR	11314	11170.43	0.01842	0.174329681	811	921.533	-0.1843	0.00027145
	Coding (non-synonymous)	3452	3778.018	-0.1302	1.13246E-07	233	196.859	0.24316	0.00999997
	Coding (synonymous)	4105	4392.445	-0.0976	1.44361E-05	147	228.875	-0.6387	6.2347E-08
	Downstream	15638	15621.36	0.00154	0.894098875	929	813.975	0.19069	5.538E-05
0.6-0.7	Intergenic	110658	110466.3	0.0025	0.564172143	5797	5756.02	0.01024	0.58908753
	Intragenic	154976	155174.9	-0.0019	0.613563931	8210	8085.63	0.02202	0.16662577
	Upstream	54575	53824.93	0.01997	0.001224892	2764	2804.63	-0.0211	0.44295101
	UTR	7550	7695.974	-0.0276	0.096119787	207	401.011	-0.954	**3.3811E-22**
	Coding (non-synonymous)	2341	2480.642	-0.0836	0.005051739	155	153.337	0.01557	0.89314144
	Coding (synonymous)	2673	2884.074	-0.1096	8.48213E-05	94	178.274	-0.9234	2.759E-10
	Downstream	10213	10256.97	-0.0062	0.664181507	765	634.017	0.27094	1.9722E-07
0.7-0.8	Intergenic	73115	72532.07	0.01155	0.030429516	4541	4483.44	0.0184	0.39002232
	Intragenic	101568	101887.7	-0.0045	0.316576509	6383	6298.01	0.01934	0.28419528
	Upstream	35579	35341.39	0.00967	0.206255279	2178	2184.57	-0.0043	0.88827978
	UTR	4947	5053.168	-0.0306	0.135300471	128	312.353	-1.287	**1.7895E-25**
	Coding (non-synonymous)	1350	1511.793	-0.1633	3.16646E-05	134	121.128	0.1457	0.24216517
	Coding (synonymous)	1542	1757.659	-0.1889	2.68961E-07	90	140.827	-0.6459	1.8436E-05
	Downstream	6159	6250.966	-0.0214	0.244749088	615	500.839	0.29624	3.3761E-07
0.8-0.9	Intergenic	44350	44203.66	0.00477	0.48640106	3505	3541.68	-0.015	0.53765507
	Intragenic	62614	62094.02	0.01203	0.036915965	5057	4975.09	0.02356	0.24554021
	Upstream	21319	21538.32	-0.0148	0.135073272	1716	1725.69	-0.0081	0.81554
	UTR	3102	3079.583	0.01046	0.686246804	135	246.742	-0.87	**1.1298E-12**
	Coding (non-synonymous)	861	1020.274	-0.2449	6.1517E-07	81	76.6898	0.07889	0.62259003
	Coding (synonymous)	996	1186.203	-0.2521	3.34158E-08	62	89.162	-0.5242	0.00402045
	Downstream	4013	4218.632	-0.0721	0.001545742	344	317.097	0.11748	0.13084596
0.9-1	Intergenic	29114	29832.02	-0.0351	3.22217E-05	2417	2242.35	0.10821	0.00022585
	Intragenic	43741	41905.82	0.06184	**3.1075E-19**	3063	3149.89	-0.0404	0.12158264
	Upstream	14249	14535.71	-0.0287	0.017403501	1085	1092.59	-0.0101	0.81838565
	UTR	1803	2078.339	-0.205	1.54479E-09	72	156.22	-1.1175	**1.6029E-11**

Table S8. Functional categories of SNPs exhibiting marked divergence between introduced populations A and B. SNPs included are within the 95th percentile of difference in allele frequency between the two introduced populations A and B (ΔAF> 0.73, 319,274 SNPs) that also exhibit significant allele frequency difference between introduced populations by Fisher's exact test (significance threshold $p<2.5\times10^{-11}$, corresponding to $\alpha=0.05$ corrected for multiple testing across a genome size of 2 Gb).

SNP category	Number of SNPs
Coding (non-synonymous)	2,905
Coding (synonymous)	3,676
Downstream	13,491
Intergenic	99,867
Intragenic	2,37
Upstream	143,623
UTR	48,355

Table S9. Testing deviation in read depth for non-synonymous SNPs identified as candidates for selection: adaptive divergence between introduced populations A and B, and candidates for novel selection in the new lake system (directional and relaxed selection). Read depth for each category of SNPs is compared to an equally large sample taken at random from the genome-wide biallelic SNPs used in estimation of population genetic metrics in paired t-tests. Errors were normally distributed in all samples of SNPs (Shapiro test, p>0.05). The fold change (M) between candidate SNPs and the random sample was estimated as of log2fold change in observed (candidate SNPs) vs. expected (randomly sampled SNPs) read depths.

	n	read depth	read depth (random sample)	t	df	p	M
Candidate SNPs for adaptive difference between introduced population A and B	22	74	63	2.73	21	0.01	0.24
Candidate SNPs for directional selection on chr 7	14	65	59	0.11	13	0.92	0.15
Candidate SNPs for relaxed selection on chr 28	7	64	59	0.79	6	0.46	0.1

Table S10. Genes putatively of adaptive importance for introduced populations found by considering windows (5 kb) of high differentiation between the two introduced populations simultaneously showing low diversity within each introduced population (F_{ST} above 95th percentile and diversity within each introduced population below 5th percentile of π). Gene model predictions are given for non-synonymous SNPs found within these windows that exhibit significant allele frequency difference between introduced populations, tested using Fisher's exact test in Popoolation2 (significance threshold $p<2.5\text{x}10^{-11}$, corresponding to α=0.05 corrected for multiple testing across a genome size of 2 Gb). SNP coordinate and the frequency of the major allele in each of the four populations is given. Predicted gene models (trout gene) are described as found in the brown trout genome. Gene model predictions (gene) and the general description of the biological function (function) found in the nearest related species (species) is also given for each gene model.

**Chr**	**Position**	**Frequency of major allele**					***Brown trout***		***Other species***		
		**Introduced population A**	**Introduced population B**	**Established population LB**	**Established population HV**	***p* (-log10)**	**Trout gene**	**Gene description**	**Gene**	**Function**	**Species & reference**
1	70548609	0.07	1.00	0.68	0.21	37	LOC115206622	cytochrome P450 2K1	*CYP2U1*	Arachidonic metabolism	Rainbow trout (*Oncorhynchus mykiss*) (Katchamart et al. 2002)
1	70551453	0.03	0.99	0.71	0.29	42					
1	70551474	0.99	0.01	0.33	0.73	42					
2	74904357	0.00	1.00	0.70	0.49	45	LOC115162910	cadherin-23-like	*CHD23*	Hearing	Zebrafish (*Danio rerio*) (Söllner et al. 2004)
2	74944283	0.97	0.03	0.36	0.51	33					
14	31522273	0.29	0.91	0.63	0.38	15	LOC115208429	integrin, beta 7	*ITGB7*	Immunity	Vertebrates (Takada et al. 2007)
14	56699003	0.06	0.97	0.76	0.47	41	LOC115207816	chromodomain helicase DNA binding protein 6	*CHD6*	Immunity	Atlantic salmon (*Salmo salar*) (Dettleff et al. 2017)
14	57378213	0.09	0.98	0.66	0.35	41	LOC115208416	RNA-binding protein 12-like	*RBM12*	RNA binding	Vertebrates (Yang et al. 2008)
19	14605425	0.23	0.99	0.81	0.59	25	sylt2	synaptotagmin-like 2a	*SYTL2*	Immunity	Atlantic cod (*Gadus morhua*) (Kleppe et al. 2013)
19	18820277	0.01	1.00	0.75	0.54	52	LOC115154100	alpha-tectorin-like	*TECTA*	Hearing	Mammals (Verhoeven et al. 1998)
22	28858862	0.04	0.99	0.71	0.38	39	LOC115158534	circadian locomoter output cycles protein kaput-like	*CLOCK*	Circadian rhythm	Atlantic salmon (*Salmo salar*), brown trout (*S trutta*) (Isorna et al. 2017)
22	29047369	0.05	1.00	0.75	0.35	39	LOC115158539	NA	unchar.	NA	NA

22	29058276	0.00	0.98	0.71	0.42	35	LOC115158545	multifunctional protein ADE2-like	*PAICS*	Purine metabolism	Zebrafish (Ng et al. 2009)
22	29058298	0.00	0.98	0.70	0.37	34					
22	29707140	0.01	1.00	0.90	0.31	45	LOC115158562	regulator of G-protein signaling 5-like	*RGS5*	Immunity	Atlantic salmon (*Salmo salar*) (Tacchi et al. 2011)
24	26309434	0.99	0.00	0.26	0.77	52	LOC115160992	titin-like	*TELT*	Muscle protein	Atlantic salmon *(Salmo salar*) (Ørnholt-Johansson et al. 2017)
24	26310645	0.97	0.31	0.62	0.79	23					
32	34056787	0.00	0.94	0.72	0.32	40	LOC115171775	adenylate cyclase type 9-like	*ADCY9*	cAMP production (intracellular signal transduction)	Mammals (Hacker et al. 1998)
32	34056910	0.02	0.99	0.64	0.33	47					

Table S11. Genes putatively shaped by relaxed selection in the new environments on chromosome 28. Gene model predictions are given for non-synonymous SNPs found within c. 1 Mb region flanking a window (5 kb) with reduced heterozygosity (H_P) in introduced populations compared to established populations (ZH_P>0 in both descendant populations and $ZH_{P<}$-4 in both introduced populations). The coordinate is given for each SNP along with the frequency of the major allele in each of the four populations. Predicted gene models (gene) are described as found in brown trout, three of which overlap (LOC115165612, LOC115165613, and LOC115165615). Gene name (gene) and the general description of the biological function (function) found in the nearest related species (species) is also given for each gene model.

	**Frequency of major allele**				***Brown trout***		***Other species***		
**Position**	**Introduced population A**	**Introduced population B**	**Established population LB**	**Established population HV**	**Gene**	**Gene description**	**Gene**	**Function**	**Species & reference**
16103241	0.01	0.99	0.67	0.36	LOC115165610	adipolin-like	LOC115165610	signaling receptor binding	
16123301	1	0.02	0.32	0.72	LOC115165611	45 kDa calcium-binding protein	LOC115165611	calcium ion binding, immunity	Rainbow trout (*Oncorhynchus mykiss*) (Porteros et al. 1997)
16158101	0.13	1	0.75	0.53	LOC115165612	homeodomain-interacting protein kinase 1-like	LOC115165612	ATP binding, immunity	Atlantic salmon (*Salmo salar*) (Zueva et al. 2018)
					LOC115165613	arf-GAP with coiled-coil, ANK repeat and PH domain-containing protein 3-like	LOC115165613	GTPase activator activity	
					LOC115165615	uncharacterized	LOC115165615	NA	
16159716	0.11	1	0.74	0.53	LOC115165612	homeodomain-interacting protein kinase 1-like	LOC115165612	ATP binding	Atlantic salmon (*Salmo salar*) (Zueva et al. 2018)
					LOC115165613	arf-GAP with coiled-coil, ANK repeat and PH domain-containing protein 3-like	LOC115165613	GTPase activator activity	
					LOC115165615	uncharacterized	LOC115165615	NA	
16160196	0.89	0.99	0.91	0.84	LOC115165612	homeodomain-interacting protein kinase 1-like	LOC115165612	ATP binding	Atlantic salmon (*Salmo salar*) (Zueva et al. 2018)
					LOC115165613	arf-GAP with coiled-coil, ANK repeat and PH domain-containing protein 3-like	LOC115165613	GTPase activator activity	
					LOC115165615	uncharacterized	LOC115165615	NA	
16160295	0.03	1	0.71	0.39	LOC115165612	homeodomain-interacting protein kinase 1-like	LOC115165612	ATP binding	Atlantic salmon (*Salmo salar*) (Zueva et al. 2018)
					LOC115165613	arf-GAP with coiled-coil, ANK repeat and PH domain-containing protein 3-like	LOC115165613	GTPase activator activity	
					LOC115165615	uncharacterized	LOC115165615	NA	
16171500	0.97	0	0.33	0.68	LOC115165613	arf-GAP with coiled-coil, ANK repeat and PH domain-containing protein 3-like	LOC115165613	GTPase activator activity	

Table S12. Genes putatively under directional selection in the new environments found on chromosome 7. Gene model predictions are given for non-synonymous SNPs found within regions of reduced variation on chromosome 7 in established populations compared to introduced populations ($ZH_{P} < -2$ in both established populations and $ZH_{P} > 0$ in both introduced populations; see Appendix 2 for details). The coordinate is given for each SNP along with the frequency of the major allele in each of the four populations. Predicted gene models (gene) are described as found in brown trout. Gene name (gene) and the general description of the biological function (function) found in the nearest related species (species) is also given for each gene model.

		**Frequency of major allele**				***Brown trout***		***Other species***		
**Chr**	**Position**	**Introduced population A**	**Introduced population B**	**Established population LB**	**Established population HV**	**Gene**	**Gene description**	**Gene**	**Function**	**Species & reference**
7	30024542	0.80	1.00	0.89	0.86	LOC115197214	junctional adhesion molecule A-like	*JAML*	Immunity	Grass carp (*Ctenopharyngodon idellus*), red drum (*Sciaenops ocellatus*) (Zhang et al. 2014; Du et al. 2015)
	30161516	1.00	0.92	0.93	0.99	LOC115197215	LHFPL tetraspan subfamily member 6 protein-like	*LHFPL6*	uncharacterized	
	30280884	0.66	1.00	0.89	0.95	LOC115197216	forkhead box protein O1-A-like	*FOXO1*	Metabolism	Rainbow trout (*Oncorhynchus mykiss*) (Conde-Sieira et al. 2018)
	30417516	1.00	0.72	0.59	0.90	LOC106602895	transcription factor Sox-19a-like	*LOC106602895*	Metabolism	Arctic charr (*Salvelinus alpinus*) (Striberny et al. 2019)
	30417713	1.00	0.71	0.71	0.87					
	30609653	0.68	0.69	0.57	0.83	LOC115197223	calcium signal-modulating cyclophilin ligand-like	*CAMLG*	Calcium mobilization	Human (*Homo sapiens)* (Bram et al. 1996)
	30886239	0.78	0.78	0.60	0.86	faxdc2	fatty acid hydroxylase domain containing 2	*FAXDC2*	Metabolism	Atlantic salmon (*Salmo salar*) (Caballero-Solares et al. 2018)
	30906606	0.76	0.73	0.57	0.86	larp1	La ribonucleoprotein domain family member 1	*LARP1*	Metabolism	Human (*Homo sapiens*) (Burrows et al. 2010)
	30948365	0.74	1.00	0.93	0.97					

31084864	1.00	0.69	0.57	0.85		RNA binding motif protein 41			
31084992	0.77	0.74	0.66	0.80	rbm41		*RBM41*	mRNA splicing	Vertebrates (Yang et al. 2008)
31090307	0.70	0.71	0.56	0.78		RNA binding motif protein 41			
31418246	0.71	0.99	0.94	0.81	LOC115197235	histone H4 transcription factor-like	*HINFP*	Transcription factor	Zebra fish (*Danio rerio*) (Swartz et al. 2014)
31464927	0.69	1.00	0.90	0.97	LOC115197236	nuclear cap-binding protein subunit 3-like	*NCBP3*	Immunity	Vertebrates (Yang et al. 2020)

References for tables S10-S12

Bram RJ, Valentine V, Shapiro DN, Jenkins NA, Gilbert DJ, Copeland NG. 1996. The Gene for Calcium-Modulating Cyclophilin Ligand (CAMLG) Is Located on Human Chromosome 5q23 and a Syntenic Region of Mouse Chromosome 13. Genomics 31:257–260. Academic Press.

Burrows C et al. 2010. The RNA binding protein Larp1 regulates cell division, apoptosis and cell migration. Nucleic Acids Research 38:5542–5553. Oxford Academic.

Caballero-Solares A, Xue X, Parrish CC, Foroutani MB, Taylor RG, Rise ML. 2018. Changes in the liver transcriptome of farmed Atlantic salmon (Salmo salar) fed experimental diets based on terrestrial alternatives to fish meal and fish oil. BMC Genomics 19:1–26. BMC Genomics.

Conde-Sieira M, Ceinos RM, Velasco C, Comesaña S, López-Patiño MA, Míguez JM, Soengas JL. 2018. Response of rainbow trout's (Oncorhynchus mykiss) hypothalamus to glucose and oleate assessed through transcription factors BSX, ChREBP, CREB, and FoxO1. Journal of Comparative Physiology A: Neuroethology, Sensory, Neural, and Behavioral Physiology 204:893–904. Springer Verlag.

Dettleff P, Moen T, Santi N, Martinez V. 2017. Transcriptomic analysis of spleen infected with infectious salmon anemia virus reveals distinct pattern of viral replication on resistant and susceptible Atlantic salmon (Salmo salar). Fish & shellfish immunology 61:187–193. Elsevier.

Du L, Feng S, Yin L, Wang X, Zhang A, Yang K, Zhou H. 2015. Identification and functional characterization of grass carp IL-17A/F1: An evaluation of the immunoregulatory role of teleost IL-17A/F1. Developmental & Comparative Immunology 51:202–211. Pergamon.

Hacker BM, Tomlinson JE, Wayman GA, Sultana R, Chan G, Villacres E, Disteche C, Storm DR. 1998. Cloning, Chromosomal Mapping, and Regulatory Properties of the Human Type 9 Adenylyl Cyclase (ADCY9). Genomics 50:97–104. Academic Press.

Isorna E, de Pedro N, Valenciano AI, Alonso-Gómez ÁL, Delgado MJ. 2017. Interplay between the endocrine and circadian systems in fishes. Journal of Endocrinology 232:R141–R159. Bioscientifica Ltd.

Kardos M, Luikart G, Bunch R, Dewey S, Edwards W, McWilliam S, Stephenson J, Allendorf FW, Hogg JT, Kijas J. 2015. Whole-genome resequencing uncovers molecular signatures of natural and sexual selection in wild bighorn sheep. Molecular Ecology 24:5616–5632.

Katchamart S, Miranda CL, Henderson MC, Pereira CB, Buhler DR. 2002. Effect of xenoestrogen exposure on the expression of cytochrome P450 isoforms in rainbow trout liver. Environmental Toxicology and Chemistry 21:2445–2451. John Wiley & Sons, Ltd.

Kjærner-Semb E et al. 2016. Atlantic salmon populations reveal adaptive divergence of immune related genes - a duplicated genome under selection. BMC genomics 17:610. BMC Genomics.

Kleppe L, Karlsen Ø, Edvardsen RB, Norberg B, Andersson E, Taranger GL, Wargelius A. 2013. Cortisol treatment of prespawning female cod affects cytogenesis related factors in eggs and embryos. General and Comparative Endocrinology 189:84–95. Elsevier Inc.

Ng A, Uribe RA, Yieh L, Nuckels R, Gross JM. 2009. Zebrafish mutations in gart and paics identify crucial roles for de novo purine synthesis in vertebrate pigmentation and ocular development. Development 136:2601–2611. The Company of Biologists.

Ørnholt-Johansson G, Frosch S, Gudjónsdóttir M, Wulff T, Jessen F. 2017. Muscle Protein Profiles Used for Prediction of Texture of Farmed Salmon (Salmo salar L.). Journal of Agricultural and Food Chemistry 65:3413–3421.

Porteros A, Arévalo R, Weruaga E, Crespo C, Briñón JG, Alonso JR, Aijón J. 1997. Calretinin immunoreactivity in the developing olfactory system of the rainbow trout. Developmental Brain Research 100:101–109. Elsevier.

Söllner C et al. 2004. Mutations in cadherin 23 affect tip links in zebrafish sensory hair cells. Nature 2004 428:6986 428:955–959. Nature Publishing Group.

Striberny A, Jørgensen EH, Klopp C, Magnanou E. 2019. Arctic charr brain transcriptome strongly affected by summer seasonal growth but only subtly by feed deprivation. BMC Genomics 20:1–22. BMC Genomics.

Swartz ME, Wells MB, Griffin M, McCarthy N, Lovely CB, McGurk P, Rozacky J, Eberhart JK. 2014. A Screen of Zebrafish Mutants Identifies Ethanol-Sensitive Genetic Loci. Alcoholism: Clinical and Experimental Research 38:694–703. John Wiley & Sons, Ltd.

Tacchi L, Bron JE, Taggart JB, Secombes CJ, Bickerdike R, Adler MA, Takle H, Martin SAM. 2011. Multiple tissue transcriptomic responses to piscirickettsia salmonis in atlantic salmon (salmo salar). Physiological Genomics 43:1241–1254.

Takada Y, Ye X, Simon S. 2007. The integrins. Genome Biology 8:1–9. BioMed Central.

Verhoeven K et al. 1998. Mutations in the human alpha-tectorin gene cause autosomal dominant non-syndromic hearing impairment. Nature genetics 19:60–62. Nat Genet.

Willoughby JR, Harder AM, Tennessen JA, Scribner KT, Christie MR. 2018. Rapid genetic adaptation to a novel environment despite a genome-wide reduction in genetic diversity. Molecular Ecology 27:4041–4051.

Yang W, Ng P, Zhao M, Wong TKF, Yiu SM, Lau YL. 2008. Promoter-sharing by different genes in human genome - CPNE1 and RBM12 gene pair as an example. BMC Genomics 9:1–16. BioMed Central.

Yang Z, Pan Y, Chen T, Li L, Zou W, Liu D, Xue D, Wang X, Lin G. 2020. Cytotoxicity and Immune Dysfunction of Dendritic Cells Caused by Graphene Oxide. Frontiers in Pharmacology 11:1206. Frontiers Media S.A.

Zhang J, Zhang M, Sun L. 2014. Junctional adhesion molecule A of red drum (Sciaenops ocellatus): A possible immunomodulator and a target for bacterial immune evasion. Veterinary Immunology and Immunopathology 161:99–107. Elsevier.

Zueva KJ, Lumme J, Veselov AE, Kent MP, Primmer CR. 2018. Genomic signatures of parasite-driven natural selection in north European Atlantic salmon (Salmo salar). Marine Genomics 39:26–38. Elsevier.

Table S13. Glossary for some of the terminology used.

Concept	Description
AF	Allele frequency within a population pool of the globally major allele per variant site (SNP), i.e., the allele that is most common across all four population pools analyzed in the present study.
ΔAF	Pairwise difference in allele frequency of major allele between population pools
Major allele	Most common allele found across all population pools
Minor allele	Least common allele found across all population pools
Hp	Heterozygosity score per population pool calculated as: $H_P = 2\ \Sigma n_{MAJ}\ \Sigma n_{MIN}/(\Sigma n_{MAJ} + \Sigma n_{MIN})^2$, where Σn_{MAJ} and Σn_{MIN} are the sums of the major and minor allele counts across all pools, respectively (Rubin et al. 2010)
ZHP	H_P normalized relative its mean and standard deviation as follows: $ZH_P=(H_P-\mu_{HP})/\sigma_{HP}$, where μ_{HP} and σ_{HP} are the mean and standard deviation of H_P, respectively (Rubin et al. 2010). The distribution of ZH_P is characterized by $\mu_{HP}=0$ and $\sigma_{HP}=1$
Gene model	A region of the genome predicted to be translated into a protein.
Non-synonymous	Substitutions within coding regions (regions of the genome predicted to encode proteins) that change the encoded amino acid, i.e. a missense mutation
Synonymous	Substitutions within coding regions (regions of the genome predicted to encode proteins) that do not change the encoded amino acid
Downstream	Substitutions within a 5 kb long nucleotide sequence downstream of a gene
Intergenic	Substitutions within the nucleotide sequence located between genes
Intragenic	Substitutions within the non-coding region of the nucleotide sequence within a gene (e.g. introns)
Upstream	Substitutions within a 5 kb long nucleotide sequence upstream of a gene
UTR	Either of two untranslated regions (5' and 3') that flank the coding sequence of a strand of mRNA
bp	base pair
kb	10^3 base pair
Mb	10^6 base pair
Gb	10^9 base pair
M-value	M-values equal the log2fold change of the observed number of SNPs in a given annotation category for a specific interval of *ΔAF* against the expected SNP count. M-value shows relative abundance of SNPs in a given AF with different functional annotation. Positive values show observed frequency is more than expected while negative shows observed frequencies is less than expected

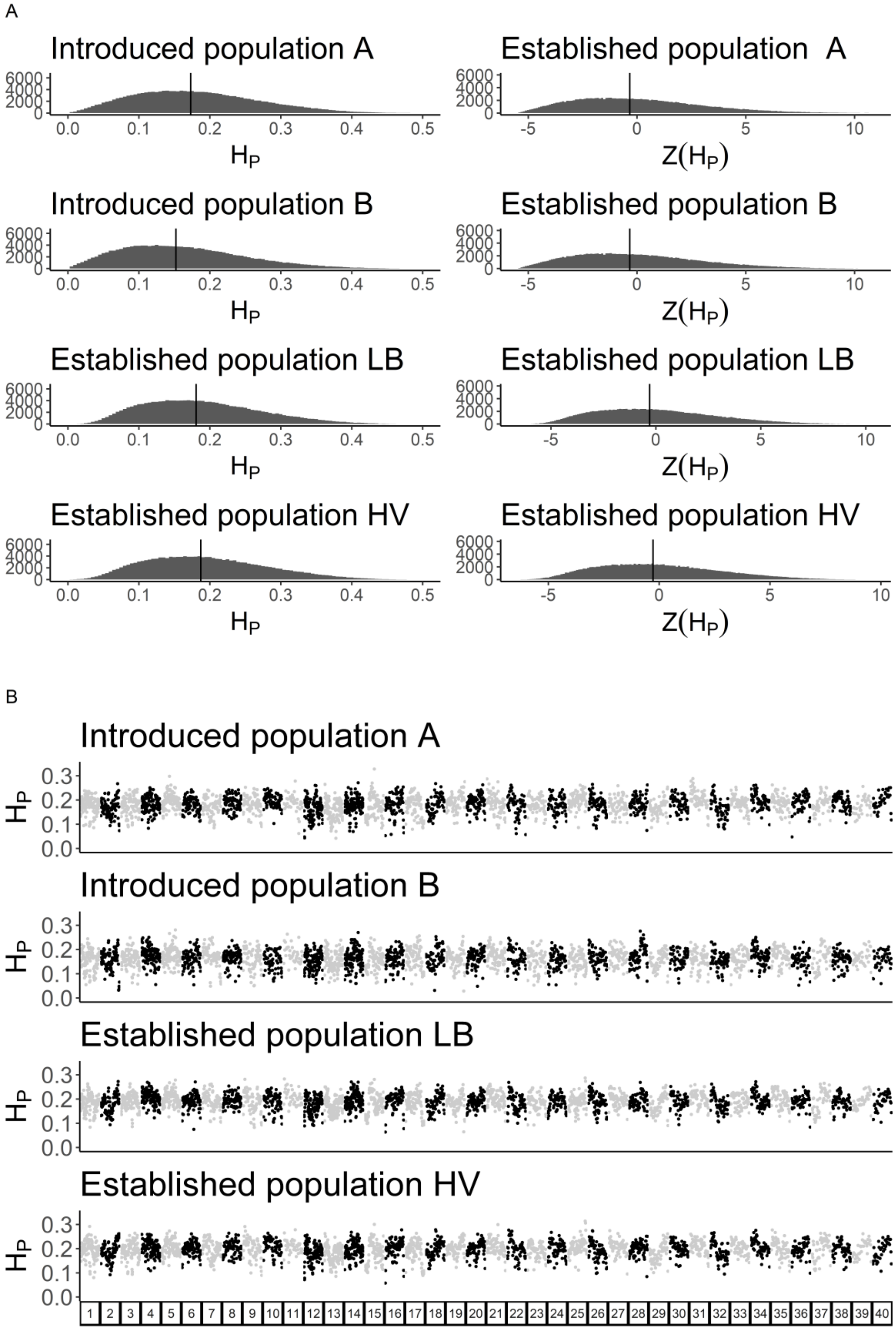

Figure S1. Genome wide heterozygosity per pool (H_P) in 5 kb windows. (A) Distributions of H_P and Z-transformed H_P (ZH_P) with median values depicted by black vertical lines and (B) Manhattan plot of H_P along chromosomal coordinates for each pool.

Pool	Mean ($x10^{-2}$)	95% CI ($x10^{-2}$)	Range ($x10^{-2}$)
Introduced population A	0.141	0.140 - 0.141	0 - 3.814
Introduced population B	0.122	0.122 - 0.123	0 - 3.745
Established population LB	0.145	0.144 - 0.145	0 - 3.719
Established population HV	0.153	0.152 - 0.153	0 - 3.58

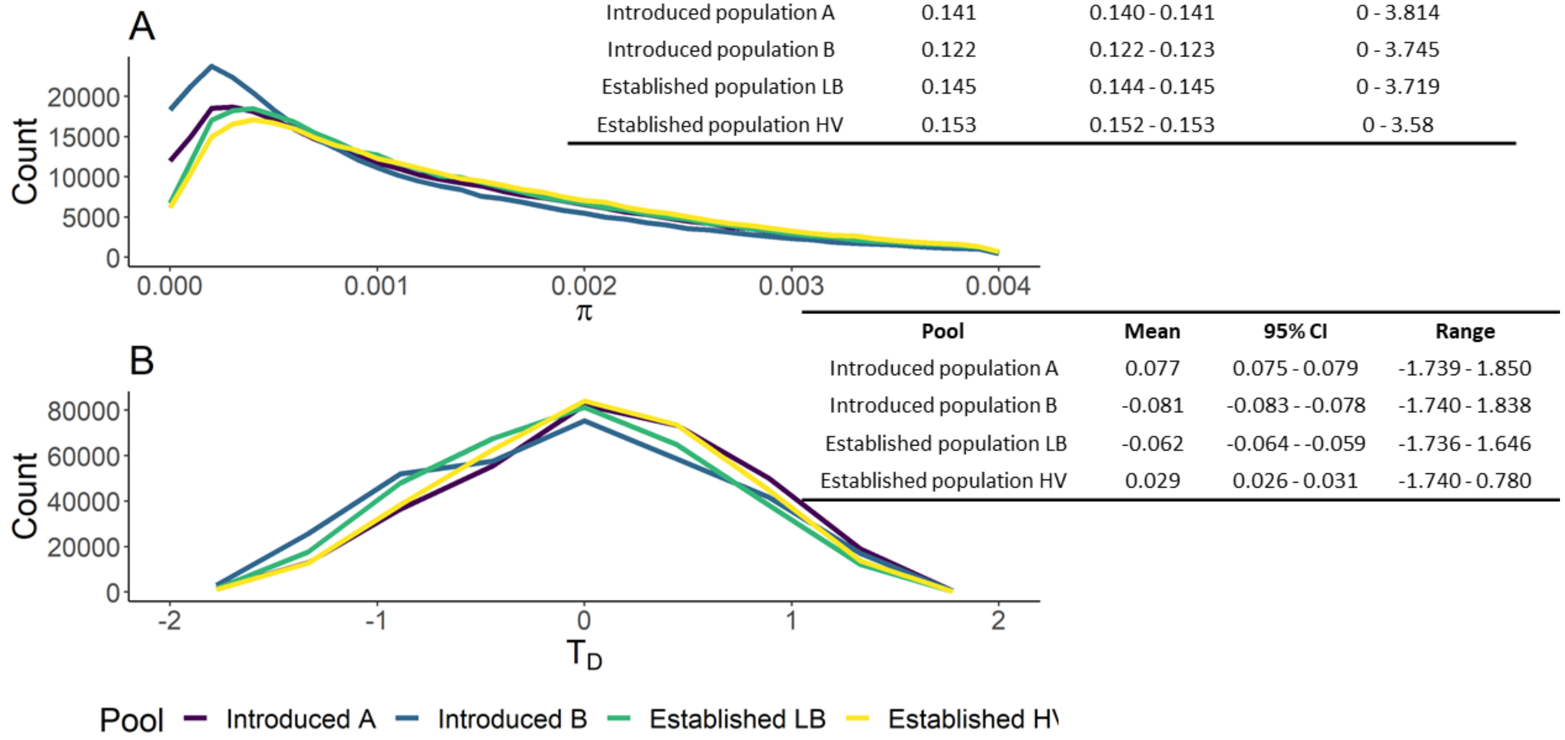

Pool	Mean	95% CI	Range
Introduced population A	0.077	0.075 - 0.079	-1.739 - 1.850
Introduced population B	-0.081	-0.083 - -0.078	-1.740 - 1.838
Established population LB	-0.062	-0.064 - -0.059	-1.736 - 1.646
Established population HV	0.029	0.026 - 0.031	-1.740 - 0.780

Figure S2. Distribution of genome wide nucleotide diversity (π) and Tajima's D (T_D) within population pools. Tables include the population mean for each measure, their 95% confidence intervals (CI), and range. π and T_D were estimated across 331,179 windows corresponding to 5,740,787 variants. Window size = 5,000 bp (5 kb) and fraction depth covered ≥ 0.8 (i.e. a window was only retained if at least 80% of its SNPs had a read depth between 20X and 150X).

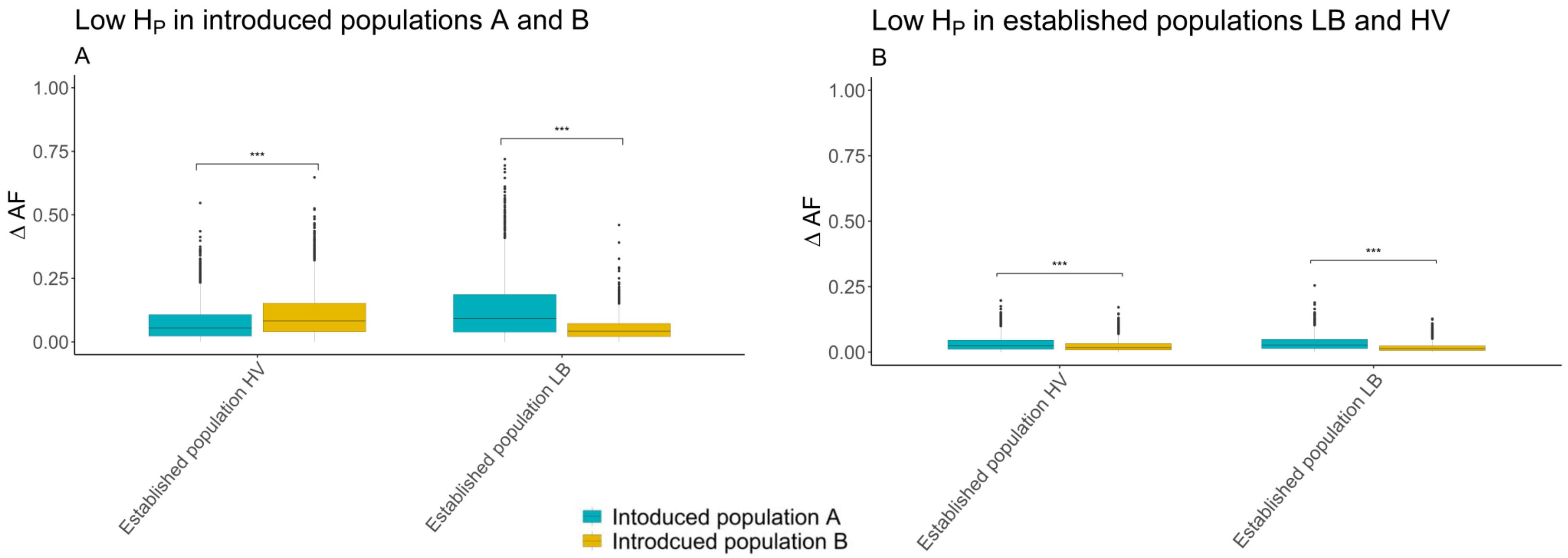

Figure S3. Boxplots of difference in allele frequency (*ΔAF*) between each of the introduced populations A and B and established populations LB and HV for regions putatively under selection in introduced populations (A) and established populations (B), respectively. (A) shows *ΔAF* for 2,106 windows (5 kb) with low genetic variation in the introduced populations as compared to the established (Z-transformed *HP* (ZH_P)>0 in both established populations and ZH_P <0 in both introduced populations) potentially indicating relaxed selection in the new environments. Each established population is compared to introduced population A (blue) and B (orange). (B) shows *ΔAF* for 1,642 windows of reduced variation in established populations as compared to introduced populations (ZH_P<0 in both established populations and ZH_P>0 in both introduced populations) potentially indicating regions of directional selection in the new environment. Each established population is compared to Introduced populations A (blue) and B (orange). Stars above brackets indicate significance levels of two-sample Wilcoxon test.

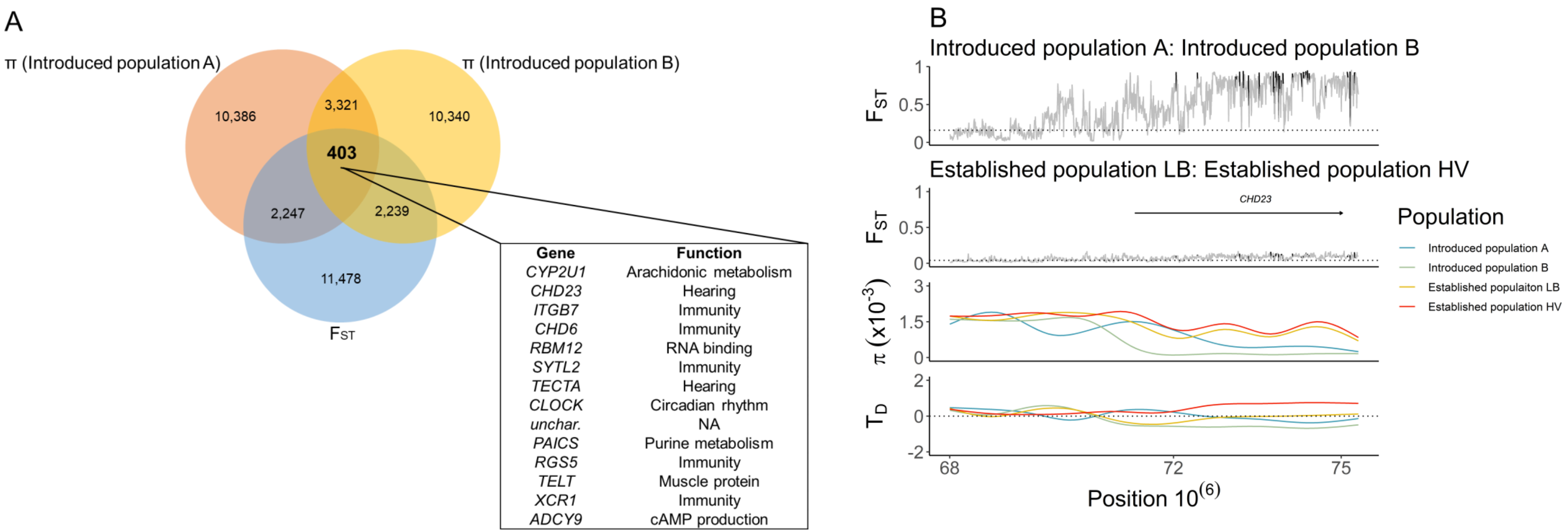

Figure S4. Potentially adaptive differences between the two introduced populations A and B. (A) Windows (5 kb) showing low diversity within each of introduced population A and B (diversity within each introduced population below 5th percentile of π; blue and yellow circles for introduced population A and B, respectively) and of marked divergence between introduced populations (F_{ST} above 95th percentile, red circle), and their overlap. Candidates for adaptive divergence are defined as windows exhibiting marked differentiation between introduced populations with simultaneously low diversity within each introduced population; 403 such windows are found and genes predicted for non-synonymous SNPs within these windows are listed (Table S8). (B) Diversity and divergence surrounding candidate for adaptive divergence; a c. 2 M base pair (Mb) region on chromosome 2 containing a swarm of fixed SNPs F_{ST} between introduced populations A and B. F_{ST} between released introduced population A and B and established populations LB and HV, and π and T_D for each population is included. Arrows refer to gene models predicted for non-synonymous SNPs within these regions (Table S8). Windows where differentiation between the released introduced populations is above the 95th percentile of F_{ST} and diversity within each introduced population below the 5th percentile of π are marked in black. F_{ST} and π were estimated within 5 kb windows.
